# Dysregulation of the Upb1-3-ureidopropionate Pathway Impairs Fatty Acid Metabolism and Exacerbates Acute Kidney Injury

**DOI:** 10.7150/ijbs.135329

**Published:** 2026-08-24

**Authors:** Yu Zhang, Jia He, Runxin Zeng, Mingzhu Jiang, Shuang Xu, Yuxian Guo, Yue Zhang, Zhanjun Jia, Ran You, Mengqiu Wu, Aihua Zhang, Mi Bai

**Affiliations:** 1 Nanjing Children's Hospital, Clinical Teaching Hospital of Medical School, Nanjing University, Nanjing, China.; 2 Nanjing Key Laboratory of Pediatrics, Children's Hospital of Nanjing Medical University, Nanjing, China.; 3 Jiangsu Key Laboratory of Early Development and Chronic Diseases Prevention in Children, Nanjing Medical University, Nanjing, China.; 4 Department of Nephrology, Children's Hospital of Nanjing Medical University, Nanjing, China.

**Keywords:** 3-ureidopropionic acid, Upb1, Meis1, pyrimidine catabolism, fatty acid metabolism, acute kidney injury

## Abstract

Acute kidney injury (AKI) is a prevalent clinical syndrome associated with high mortality and lacking effective therapies, largely due to incomplete understanding of its pathogenic mechanisms. Proximal tubular epithelial cells (PTECs), with their high metabolic demand, represent the primary targets of injury in AKI. We identified β-ureidopropionase 1 (Upb1), a key enzyme in pyrimidine catabolism, as being specifically enriched in PTECs but markedly downregulated upon AKI. Genetic silencing of Upb1 exacerbated IR-induced AKI. Targeted metabolomics revealed that loss of renal Upb1 drives accumulation of 3-ureidopropionate (3-UPA), a pyrimidine catabolic intermediate. Integrated multi-omics analyses and functional validation demonstrated that 3-UPA impairs fatty acid oxidation through DNMT1-mediated DNA methylation, leading to mitochondrial structural and functional defects. Transcription factor analysis further revealed that myeloid ecotropic viral integration site 1 (Meis1) transcriptionally represses Upb1. Tubule-specific Meis1 overexpression aggravated folic acid (FA)- and ischemia/reperfusion injury (IR)-induced AKI, whereas pharmacological inhibition of Meis1 alleviated tubular damage. Notably, Meis1 overexpression induced marked 3-UPA accumulation, reinforcing its role as an upstream regulator of this metabolic axis. Collectively, these results demonstrate that Meis1/Upb1/3-UPA axis exerted a detrimental role in AKI, and targeting this axis (inhibiting Meis1 or activating Upb1) has therapeutic potential for AKI.

## Introduction

Acute kidney injury (AKI) is a common clinical syndrome that can be induced by various factors such as ischemia and nephrotoxic drugs 1. It is associated with short-term and long-term morbidity and mortality 2. Recently, AKI has emerged as a major global public health challenge affecting both adults and children 3-5, but there are currently no effective therapies due to incomplete understanding on pathogenic mechanisms.

The kidney is a highly metabolically active organ in which efficient metabolism is not merely supportive but central to both health and disease. Its substantial energy demands, driven by excretory and homeostatic functions, render it particularly vulnerable to metabolic disturbances 6, 7. Renal tubular epithelial cells, particularly those in the proximal tubule, exhibit exceptionally high energy demands owing to their intense reabsorptive activity. Proximal tubular epithelial cells (PTECs) sustain their energetic requirements primarily through fatty acid β-oxidation, the tricarboxylic acid cycle (TCA), and oxidative phosphorylation. Glycogen metabolism and amino acid catabolism also contribute significantly to supporting proximal tubular epithelial cell function 8. Therefore, PTECs exhibit high metabolic activity and are consequently highly susceptible to injury 9-13. Tubular epithelial cell injury represents a conserved pathological hallmark and serves as the basis for AKI stemming from various etiological factors 14. Protecting tubular epithelial cells offers a potential therapeutic approach for combating AKI. Further understanding of the pathogenesis of AKI and finding effective therapeutic targets are the focus of current research.

We identified genes specifically expressed in proximal tubular epithelial cells using the National Library of Medicine (NIH) and Kidney Interactive Transcriptomics (KIT) databases, and found that β-ureidopropionase 1 (Upb1) is highly expressed in these cells within healthy adult and mouse kidneys, with expression levels significantly differing from those in other renal cell types. Interestingly, expression of Upb1 was markedly reduced in the kidney following AKI. The Upb1 gene encodes the enzyme responsible for the final step in the pyrimidine degradation pathway. Pyrimidine nucleotides are critical for nucleic acid synthesis, glycosylation, phospholipid formation, cellular biomass, and homeostasis 15. Recent evidence shows that they promote de novo lipogenesis by sustaining mitochondrial pyruvate oxidation and TCA cycle activity, highlighting new therapeutic avenues for obesity and metabolic disorders 16. However, current research has primarily focused on pyrimidine biosynthesis, while Upb1-mediated pyrimidine catabolism has been largely overlooked, and its role in AKI remains unexplored.

Under physiological conditions, dihydropyrimidine dehydrogenase (DPYD) and dihydropyrimidinase (DPYS) catalyze the conversion of uracil into 3-ureidopropionate (3-UPA). Subsequently, Upb1 degrades over 85% of 3-UPA into water, carbon dioxide, and β-alanine, while a portion re-enters the pyrimidine metabolic cycle; only about 7% is excreted in urine 17. Studies have found that in certain disease states, the content of 3-UPA in body fluids changes 18-20, and mutations in the Upb1 gene can cause pathological accumulation of 3-UPA 21, 22. The role of 3-UPA in diseases remains unclear, with only one study reporting its potential toxicity in chicken neuronal cells 23. Nevertheless, no research has yet investigated whether 3-UPA contributes to kidney injury.

To investigate the mechanisms driving the downregulation of Upb1 expression during AKI, we queried transcription factor analysis databases Genomatix (http://www.genomatix.de) and JASPAR (http://jaspar.genereg.net) for potential regulators of the Upb1 promoter. This analysis identified several putative binding sites for Myeloid ecological viral integration site 1 (Meis1) within the promoter region. Meis1 is a member of the homologous domain transcription factor three amino acid loop extension (TALE) family and has garnered significant interest in recent years due to its role in regulating cell oxidative stress, cell cycle, proliferation and differentiation 24. Furthermore, Meis1 holds significant importance in cellular metabolism. The suppression of Meis1 hinders the expression of hypoxia inducible factor (HIF)-1α and HIF-2α, crucial regulators of glycolysis 25. Meis1 could potentially play a role in reprogramming glycerol metabolism as well 26. Previously, we reported that Meis1 in fibroblasts contributed to the pathogenesis of chronic kidney disease (CKD) 27. However, no reports have demonstrated the involvement of Meis1 in AKI.

In the present study, we have used human renal biopsy samples, a variety of AKI animal models, as well as proximal tubular epithethial cells, to demonstrate the role of Meis1/Upb1/3-UPA axis in the pathogenesis of AKI. Additionally, we conducted a comprehensive analysis of transcriptomics and metabolomics to unravel the mechanism involved.

## Materials and Methods

### Reagents and antibodies

Fetal bovine serum (FBS, Cat No. 10091148), Dulbecco's modified Eagle's medium (DMEM)-F12 (Cat No. C11995500BT), insulin (Cat No. P3376-400IU) and 0.25% trypsin-0.02% EDTA (Cat No. 25200-056) were obtained from Gibco (Waltham, MA, USA). Antibodies against Meis1 (Cat No. ab19867) and NGAL (Cat No. ab63929) were purchased from Abcam (Cambridge, MA, USA). Antibody used to detect Meis1 expression in cytoplasm and nucleus by Western blot assay were purchased from Origene (Cat No. TA809619S). Anti-Caspase 3 (Cat No. 14220s) were from Cell Signaling Technology (Boston, MA, USA). Anti-Upb1 (Cat No. A15452 for Western blot), DNMT1 (Cat No. A19679), DNMT3b (Cat No. A22658) and α-tubulin (Cat No. AC012) were from Abclonal (Wuhan, China). Anti-Flag (Cat No. F1804), anti-Upb1 (Cat No. HPA076321 for human IHC) and 3-UPA (Cat No. 94295) were obtained from Sigma-Aldrich (St. Louis, MO, USA). Meis1 inhibitor MEISi (Cat No. HY-134259), 3-UPA-FITC (Cat No. HY-113285), Lamin B1 antibody, DNMT3a antibody (Cat No. HY-P80107) and (Cat No. HY-P80205) were from MedChemExpress (Monmouth Junction, NJ, USA). Anti-KIM1 (Cat No. AF1817) was from R&D Systems (Minneapolis, MN, USA). Anti-GAPDH (Cat No. 6004-1-IG) and OXPHOS Cocktail (Cat No. PK30006) were from Proteintech Group (Rosemont, IL, USA).

### Patients

Renal biopsy specimens were procured from patients diagnosed with AKI who were undergoing diagnostic evaluation at the Department of Nephrology, Children's Hospital of Nanjing Medical University. The inclusion criterion for biopsy samples was the presence of a minimum of ten glomeruli in paraffin-embedded tissue suitable for histological sectioning. A cohort of ten subjects, aged between 4 months and 13 years, was enrolled in the study, with the majority receiving a pathological diagnosis of acute tubular necrosis. Detailed patient information is provided in Table S1. Normal renal tissues were obtained from patients without proteinuria who underwent partial nephrectomy for benign renal tumors.

The protocol for the utilization of biopsied samples and nephrectomized tissues was approved by the local ethics committee on human subjects at Children's Hospital of Nanjing Medical University (ethical approval number: 202309001-1). Written informed consent was obtained from each patient and their parents.

### Animals

**Generation of Upb1^+/-^ mouse strain**: Upb1-KO mice (Strain NO. T035101, genetic background: C57BL/6J) were purchased from GemPharmatech (Nanjing, China). Heterozygous F0 generation mice were obtained by freezing sperm resuscitation. After hybridization of F0 generation mice with WT mice (C57BL/6J), a sufficient number of F1 generation heterozygous mice (Upb1^+/-^) were obtained for experiment (8 weeks old, male).

**Generation of a renal proximal tubular** ***Meis1***** Conditional Knockin (cKI) Mouse Strain**: The Meis1*^flox/flox^* knock-in mice were purchased from GemPharmatech. Using the CRISPR system, insert the transcription termination signal original STOP between the pCAG promoter and Meis1 sequence, construct the pCAG-loxp-STOP-loxp-Meis1 skeleton vector and transfer it into the male pronucleus of the fertilized egg to construct the transgenic mice, and then crossed with the *Kap*-Cre transgenic mice to generate F1. Then, F1 mated backcross with the *Meis1^flox/flox^* knock-in mice to generate renal proximal tubular *Meis1* conditional knock-in (Meis1*^flox/flox^*; Kap-Cre^+^, cKI) mice. The wide type (Meis1^flox/flox^; Kap-Cre^-^, WT) from the same litters were used as controls.

**Bilateral ischemia/reperfusion injury (IR) model**: In male mice aged 8-10 weeks, non-invasive miniature arterial clips were used to quickly occlude right and left renal pedicles. The kidney turned from red to purple black, indicating successful occlusion, for a total of 30 minutes. After releasing the arterial clip, the kidney changed from purple black to red. The control group mice opened their abdominal cavities, but did not block the renal pedicle. During the experiment, body temperature of mice was controlled at 36.5 °C-37.5 °C. Mice were sacrificed after 24 or 48 hours.

**Folic acid (FA) model:** According to previous studies 28, kidney injury was induced by injecting intraperitoneally 250 mg/kg of FA dissolved in 0.3 M sodium bicarbonate (NaHCO3). Control mice were intraperitoneally (i.p.) injected with the same amount of 0.3 M NaHCO3 (Vehicle). Mice were sacrificed after 48 hours.

**3-UPA treatment:** Wild type C57BL/6J mice (8 weeks old, male) were obtained from GemPharmatech (Nanjing, China). For intervention of IR model, the mice were treated with 3-UPA at 180 mg/kg/day via i.p. injection after IR surgery. Then the mice were treated twice a day for 2 consecutive days and sacrificed. For intervention of FA, the mice were treated with 3-UPA at 180 mg/kg/day or 260 mg/kg/day via i.p. injection after FA delivery. The mice were treated twice a day for 2 consecutive days and sacrificed.

**High-throughput tail vein plasmid delivery:** According to our previous study 27, Upb1 or Vehicle plasmids and the vehicle were dissolved in saline to a working concentration of 35μg mL^-1^ before injection. Then injected 2mL of the plasmids to the mice through the tail vein via high-throughput within 10 seconds. After 24 hours, the mice were conducted with FA model and 3-UPA treatment.

**Meis1 inhibitor (MEISi) treatment:** C57BL/6J mice (8 weeks old, male) were purchased from GemPharmatech (Nanjing, China). For intervention of FA or IR model, the mice were treated with MEISi at 0.4 mg/kg/day via i.p. injection 24 h before FA deliduvery or IR surgery. Then the mice were treated daily for 2 consecutive days and sacrificed.

All mice were maintained in a temperature-controlled room (19-21 °C) on a 12-h light-dark cycle, fed a standard rodent diet, and had free access to water. All animal procedures were approved by the Institutional Animal Care and Use Committee at Nanjing Medical University, China (ethical approval number: IACUC-2310044).

### Biochemical analysis

Blood samples were collected from the mice and centrifuged at 1500×g to obtain serum. The biochemical parameters for kidney function, such as serum creatinine (Scr) and blood urea nitrogen (BUN), were tested using automatic biochemical analyzers at Children's Hospital of Nanjing Medical University.

### Tissue fixation, section, and staining

The kidney tissues were fixed overnight in 4% paraformaldehyde, dehydrated, embedded in paraffin, and cut into 3 μm sections. After deparaffinisation, the kidney sections were stained with Periodic Acid-Schiff (PAS) staining as previously reported 28. The tubular injury score was performed by a trained and blinded experimenter.

Terminal deoxynucleotidyl transferase-mediated dUTP nick end labeling (TUNEL) staining was performed according to the manufacture's protocol (Cat No. A112, Vazyme, China). Cellular apoptosis was imaged by confocal microscopy (LSM710, Carl Zeiss, Germany), analyzed with Image J, and quantified as previously described 29.

### 3-UPA detection

We used ultra-high performance liquid chromatography-tandem mass spectrometry (UHPLC-MS/MS) target metabolomics to detect 3-UPA content in tissues or cells. Firstly, metabolite extraction was carried out. An aliquot of each individual sample was precise weighed and were transferred to an Eppendorf tube. After the addition of 1000 μL of extract solution (methanol-acetonitrile-water, 2:2:1, radio of volume, precooled at -40 °C), the samples were vortexed for 30 s and sonicated for 5 min in the ice-water bath, and homogenized at 40 Hz for 4 min. The homogenate and sonicate circle were repeated three times. After incubation at -40 °C for one hour, the samples were centrifuged at 12000 rpm (RCF = 13800(×g), R= 8.6cm) for 15 min at 4 °C. A 100 μL aliquot of the supernatant and the diluent were subjected to UHPLC-MS/MS analysis (supported by Shanghai Biotree Biomedical Technology CO., LTD).

### Cell culture

The mouse proximal tubule epithelial cells (TKPTs, purchased from ATCC, cat: CRL-3361) was maintained in DMEM-F12 medium containing 7% FBS and 6 mg/L insulin. All cells were cultured in a humidified incubator with 5% CO_2_ at 37 °C. The cells were transfected with Meis1 or Upb1 plasmids using jetPRIME (cat: 114-15, Polyplus, Strasbourg, France) according to the manufacturer. Then the cells were placed in a three-gas incubator (37 °C, 5%CO_2_, 1%O_2_, 94%N_2_) for anoxic culture for 24 hours and reoxygenated for 2 hours (hypoxia/reoxygenation, H/R). In certain experiments, the cells were treated with MEISi (1μM) or 3-UPA (0.1-10mM).

### RNA-sequencing

Total RNA was extracted from TKPTs after stimulation with 3-UPA for 24 hours. RNA sequencing libraries were prepared and sequenced by Annoroad Gene Technology Co., Ltd. (Beijing, China). Sequencing was performed on the DNBSEQ platform using the T7 sequencing chemistry, following the manufacturer's standard protocols.

### DNA methylation detection

**Total DNA methylation:** the degree of 5-mC methylation in total DNA samples from TKPTs was measured using the MethylFlash Global DNA Methylation (5-mC) ELISA Easy Kit (Epigentek, P-1030-48). In brief, genomic DNA was extracted from the TKPTs using Universal Genomic DNA Purification Mini Spin Kit (Cat No. D0063, Beyotime, Beijing, China). Then follow the steps of the ELISA kit, and finally read the absorbance 450 on the microcoder (Molecular Devices, CA, USA). Finally, according to the standard curve, calculated the percentage of methylated DNA (5-mC) in total DNA.

**Reduced representation bisulfite sequencing (RRBS):** Genomic DNA was extracted by Qiagen DNeasy® Blood & Tissue kit (Qiagen, Hilden, Alemanha) from cell pellet. Qubit dsDNA HS assay kit (Sangon, Shanghai, China) was used to test concentration and 1% Agarose gel electrophoresis to confirm integrity. The library preparation and next generation sequence was finished by Sangon Biotech (Shanghai) Co., Ltd. Briefly, genomic DNA was treated by MspI enzyme (NEB) -which cuts DNA at all CCGG sites, regardless of their DNA methylation status at the CG site followed by adaptor ligation. Then bisulfite was added to convert the unmethylated cytosines in the DNA fragment to uracil while the methylated cytosines were not affected. Then PCR amplification was performed to complete the library preparation. Purified libraries were assessed for quality and quantity using Qubit 4.0 (Thermo, Waltham, USA) and 2% agarose gel electrophoresis respectively. Finally, the libraries were pooled and loaded on Novaseq 6000 (Illumina, San Diego, USA) sequencer by 2×150bp paired end sequence kit according to the manufacturer's instructions.

### Quantitative real-time PCR

According to our previous report 27, total RNA from tissues or cells was extract using RNA isoPlus reagent (cat: 9109, TaKaRa Biotechnology, Japan). cDNA was reverse transcribed with HiScript II Q RT SuperMix for qPCR (cat: R222-01, Vazyme Biotechnology, Nanjing, China). Real-time PCR amplification was carried out through the LightCycler®96 (Roche) real-time PCR detection system using AceQ qPCR SYBR Green Master Mix (cat: Q131-02, Vazyme Biotechnology). The temperature cycling conditions were 95 °C for 10 min followed by 40 cycles of 95 °C for 15 s and 60 °C for 1 min. The relative expression level of mRNA was normalized to the relative expression of GAPDH and calculated using the 2 ^-ΔΔCt^ method. The primers were designed by Primer 5.0 software (http://Frodo.wi.mit.edu) and the primer sequences are shown in Table S2.

### Western blot

Tissue or cell lysate was homogenized with RIPA buffer containing 1×protease inhibitor (cat: 04693132001, Roche, Basel, Switzerland) and 1×phosphatase inhibitor (cat: 04906837001, Roche). The samples were then centrifuged at 5000 rpm for 30 min and the protein concentration was determined using a BCA protein detection kit (cat: 23227, ThermoFisher). Equivalent samples were used for primary antibodies against KIM1 (1:3000), NGAL (1:2000), Meis1 (1:1000), Caspase 3 (1:1000), Upb1 (1:1000), Lamin B1 (1:1000), α-tubulin (1:1000), Flag (1:1000), OXPHOS Cocktail (1:1000), DNMT1 (1:1000), DNMT3a (1:5000), DNMT3b (1:5000) and GAPDH (1:2000), and then added HRP-labelled secondary antibody (1:5000). The immunoreactive bands were visualized using the Tanon 5200 Multi-function imaging system. Density analysis is performed by measuring the band intensity and using Image J software to normalize it to the corresponding GAPDH band.

### Immunohistochemistry (IHC)

The kidney slides (3µm thick) were dewaxed and rehydrated with xylene and graded ethanol by standard method. Slides were incubated in 3% hydrogen peroxide for 20 minutes and boiled in improved Citrate Antigen Retrieval Solution (Beyotime, Cat No. P0086, Beijing, China) for 20 minutes. The slides were then incubated overnight with primary antibodies against Meis1(1:400) and Upb1(1:500) at 4 °C. Biotin-conjugated secondary antibodies were applied after washing with phosphate buffer saline. The chromogenic reaction was carried out with diaminobenzidine, and the nucleus was backstained with hematoxylin. We finally used Image ProPlus for image analysis and quantification.

### Immunofluorescence staining

The kidney slides (3µm thick) were dewaxed and rehydrated with xylene and graded ethanol by standard method. After penetration with 0.1% Triton X-100, primary antibody Meis1 (1:1000) and LTL (1:400) were added to the slide and incubated at 4 °C overnight. Goat Anti-Rabbit IgG H&L (Alexa Fluor®555) and goat anti-mouse IgG H&L (Alexa Fluor®488) were used after washing with PBST. The nucleus was stained with DAPI. Finally, the slides were observed under a laser scanning confocal microscope (Zeiss), photographed and recorded.

TKPTs were cultured on a glass-bottom cell culture dish (NEST, China) and then treated with different concentrations of 3-UPA-FITC (10mM) for 24 h. Then, after several washes with PBS, the cells were viewed under a laser scanning confocal microscope (Zeiss), photographed and recorded.

### Oil red O staining

The kidneys of AKI model mice were used for cross-sections. The collected kidney tissue was embedded in the embedding agent optimal cutting temperature (OCT) compound to prepare frozen slices (10 µm) at -20 °C. Rinse slices slightly with distilled water for 2 min 3 times. Soak sections in 60% isopropanol for 20-30 s. Then, stain section in Modified Oil Red O Stain Solution (Solarbio, G1261) for 1 h. Then, the slices were washed slightly in 60% isopropanol to remove the dye solution and for 1 min in running water. The slices were re-dyeing by Mayer's Hematoxylin Solution for 2 min and washed by tap water for 10 min. The dyed slices were sealed with glycerol diluted with PBS (8:2) and photographed. The degree of tissue steatosis was measured on digital microphotographs using Image-Pro Plus software.

TKPTs were pre-treated with 3-UPA (10 mM) at 60% confluency. After hypoxia/ reoxygenation model, remove the cell culture medium, wash it twice with PBS, and fix it with ORO Fixative (Solarbio, G1262) for 20 min. Then discard the ORO Fixative and wash it twice with distilled water. Add 60% isopropanol and soak for 20-30s. After discarding 60% isopropanol, add the newly prepared oil red O dyeing solution and dip it for 1h. Discard the staining solution and rinse with 60% isopropanol for 30s until the stroma is clear. Wash with water 3 times until there is no excess dye. Add Mayer hematoxylin staining solution and counterstain the nucleus for 2 min. After discarding the dye solution, wash it with water for 3 times. Add oil red O buffer for l min and discard it. The cells were covered with distilled water and observed under a microscope (MSHOT, MDX10).

### Oxygen consumption rate (OCR)

As we previously described 30, the OCR was measured using the Seahorse XF-96 Extracellular Flux Analyzer (Agilent Seahorse Bioscience, Copenhagen, Denmark). Briefly, TKPTs were treated with 3-UPA for 24 h or 48 h. On the day of the experiment, cell medium was replaced by Seahorse assay medium and cells were cultured in a CO_2_-free incubator at 37 °C for 1 h prior to evaluate basal OCR. Basal oxygen consumption was recorded for 20 min, and OCR measurements were performed over time after the successive addition of mitochondrial inhibitors: Oligomycin (1 μM), FCCP (1 μM), rotenone (0.5 μM) and antimycin A (0.5 μM). The XF-96 instrument automatically injects the compound into each well. The cell bioenergetics parameters were measured as follows: basal respiration, ATP production, maximal respiration and spare capacity.

### Transmission electron microscopy (TEM)

To evaluate the mitochondrial morphology in TKPTs and mouse renal cortex, after processing the specimen as required, add 2.5% glutaraldehyde fixative, fix at room temperature for half an hour, then transfer to 2-8 °C for cold storage and fixation. KingMed Diagnostics provides assistance for slide preparation. The sections were examined in an electron microscope (JEOL JEM-1400; Tokyo, Japan).

### Succinate dehydrogenase (SDH) staining

According to our previous report, frozen tissue sections with 10 μm thickness were prepared and stained with the SDH activity kit of GENMED SCIENTIFICS (cat: GMS80042.2, Shanghai, China). Add 50μL GENMED cleaning solution to cover the entire surface of the sample, remove the cleaning solution, add 50μL GENMED working solution, and incubate in 37 °C incubators for 10 min in the dark. After washing with cleaning solution for three times, add fixing solution 50 μL and let stand at room temperature for 15 minutes. After wash for three times using cleaning solution, mount the slides with neutral gum and photographed under the microscope.

### Luciferase reporter assay

According to our previous report 27, the Meis1 or the control plasmids were transfected into TKPTs, followed by transfection with the pGL4.19-Upb1 promoter and pRL-TK plasmids (Public Protein/Plasmid Library, China). Luciferase assays were detected using the reporter assay system (Cat No. E1910, Promega, Madison, WI, USA) in accordance with the manufacturer's protocol. Relative luciferase activity was calculated as the ratio of firefly to Renilla luciferase activity (F/R).

### Chromatin immunoprecipitation assay (ChIP) assay

According to our previous report 27, cross-linked chromatin was prepared from HEK-293T cells and enzymatic detection was performed using the SimpleChIP Enzymatic Chromatin IP Kit (Magnetic Beads) according to the manufacturer's protocol. In simple term, each ChIP reaction was immunoprecipitation with 20 μg cross-linked chromatin (cut by ultrasound and micrococcal nuclease into fragments of 300-450 bp) and 2 μg Meis1 or positive control protein Histone H3 or negative rabbit IgG control antibody. Using JASPAR (http://jaspar.genereg.net/) online software, analyzed the Upb1 transcription start site - 2000 ~ + 100 bp upstream sequence, finding potential transcription factor binding sites. ChIP primers are then designed to amplify the Upb1 promoter region and the primers listed in Table S3.

### Cleavage under targets and release using nuclease (CUT&RUN)

According to the manufacturer's protocol of CUT&RUN Assay Kit (Cat No. HD101, Vazyme Biotechnology, Nanjing, China), TKPTs were collected at room temperature, and 10^5^ cells were placed in a 1.5mL EP centrifuge tube. The cells were resuspended in Wash Buffer. Incubated with processed ConA Beads Pro for 10 minutes. After the supernatant has been discarded, a pre-cooled antibody Buffer was added and left for overnight at 4 °C. The supernatant was discarded and washed by adding Dig-wash Buffer and repeated twice. The pG-MNase Enzyme premixed liquid was added and incubated with the magnetic beads-cells complex at 4 °C for 1 h. The supernatant was discarded and washed by adding Dig-wash Buffer and repeated twice. CaCl_2_ premixed liquid was added, inverted up and down several times, and incubated with the bead-cell complex on ice for 1.5 h. Stop Buffer was added to the fragmented product and left at 37 °C for 20 minutes. Then, the chromatin-enriched product was collected into a new EP tube by centrifugation at 12,900 rpm for 5 min at 4 °C. DNA extraction was performed to obtain the target gene fragments. qPCR quantitative detection involves 3 reaction steps. Stage1: 95 °C for 30s; Stage2: 40 cycles of 95 °C for 10s and 60 °C for 30s. Stage 3: 95 °C for 15s, 60 °C for 60s and 95 °C for 15s. The relative expression level of mRNA was normalized to the relative expression of Spike in DNA and calculated using the 2^ -ΔΔCt^ method. Sequences of CUT&RUN primers were listed in Table S4.

### Nuclear and cytoplasmic extraction

According to our previous report 27, nuclear and cytoplasmic extraction solution was prepared using NE-PER^TM^ nuclear and cytoplasmic extraction reagent (Cat No. 78833, ThermoFisher) in accordance with the manufacturer's protocol using 30 mg fresh tissue.

### Untargeted metabolomics analysis

The kidneys of WT or Meis1 cKI mcie after IR model were harvested. Untargeted metabolomics analysis was conducted at BGI (Shenzhen, China), using highresolution mass spectrometer Q Exactive (Thermo Fisher Scientific, USA) to collect data from both positive and negative ions. LC-MS/MS data processing was performed using The Compound Discoverer 3.1 (Thermo Fisher Scientific, USA) software, mainly included peak extraction, peak alignment, and compound identification. Data pre-processing, statistical analysis, metabolite classification annotations and functional annotations were performed using the metabolomics R package metaX and the metabolome bioinformatic analysis pipeline.

### Statistical analysis

All data are expressed as mean ± standard error of the mean (SEM) using GraphPad Prism (version 8.0, GraphPad Software, SanDiego, CA). One-way ANOVA or two-tailed Student's t-test was used to compare differences between different groups. Pearson correlation analysis was also used. P < 0.05 was considered statistically significant. All statistical details regarding P-value were shown in the figures and n can be found in figure legends.

## Results

### Upb1 is downregulated in AKI and its overexpression suppresses tubular epithelial cells apoptosis

In our preliminary analysis using the single-cell transcriptomic database Kidney Interactive Transcriptomics (https://humphreyslab.com/SingleCell/), we identified genes specifically expressed in proximal tubular epithelial cells. We found that Upb1 is highly and specifically expressed in proximal tubular epithelial cells of both healthy human 31 (Fig. S1) and mouse 32 (Fig. 1A) kidneys, with markedly lower expression in other renal cell types. In the proximal tubule datasets of two acute kidney injury to chronic kidney disease (AKI-to-CKD) transition models 33—unilateral IR and unilateral ureteral obstruction (UUO)—Upb1 was also highly expressed in healthy samples but markedly decreased in the acutely injured kidneys (Fig. 1B).

To verify the association between Upb1 and AKI, we performed IHC staining using kidney tissue samples from 10 patients with AKI. Our findings revealed a notable decrease in UPB1 levels in the kidneys of AKI patients compared to those in healthy controls (Fig. 1C and 1D). RNA sequencing of total RNA from 20 human tissues 34 shows that UPB1 is primarily expressed in the liver and kidney, then we examined the expression of Upb1 mRNA in the livers and kidneys of mice with bilateral IR-induced AKI. Our observations revealed a notable reduction in Upb1 expression specifically in the kidneys, while there was no change in the livers (Fig. 1E and 1F). We further verified this trend in FA-AKI mice, where we detected a significant decrease in both Upb1 mRNA and protein levels in the kidneys (Fig. 1G -1I). To confirm the alterations in Upb1 expression in our proximal tubular epithelial cell line TKPTs following AKI, we constructed H/R injury models with varying hypoxia exposures. After a 24-hour hypoxic period, Upb1 protein expression dropped by 61%, and further decreased with prolonged hypoxia (Fig. 1K and 1L). This finding was corroborated by changes in Upb1 mRNA expression (Fig. 1J). Collectively, these results implied that Upb1 expression in the kidney underwent significant downregulation in AKI models.

Subsequently, to preliminarily explore the role of Upb1 in AKI *in vitro*, we constructed an Upb1 overexpression plasmid. As shown in Figure 1M and 1N, this plasmid markedly elevated Upb1 protein levels in TKPTs. Meanwhile, we discovered that overexpressing Upb1 notably counteracted the hypoxia-induced expression of cleaved Caspase 3 and mitigated tubular epithelial cells apoptosis (Fig. 1O-1Q).

### The function of Upb1 in IR-induced AKI

To further validate the role of Upb1 in AKI, we generated global Upb1^+/-^ mice and verified the down-expression of Upb1 protein in kidneys (Fig. 1V and 1W). Under baseline conditions, no significant differences were observed between Upb1^+/-^ and WT mice in serum creatinine (Scr), blood urea nitrogen (BUN), or renal morphology identified by Periodic acid-Schiff (PAS) staining (Fig. S2). We then established the IR model using Upb1^+/-^ and WT mice. Compared with WT mice, Upb1^+/-^ mice showed a significant deterioration of renal function, with further increases in the levels of Scr (Fig. 1R) and BUN (Fig. 1S). PAS staining revealed significantly aggravated pathological damage, manifested as further dilation of renal tubules, disappearance of the brush border, shedding of renal tubular cells, and formation of casts (Fig. 1T). The tubular injury score was significantly elevated (Fig. 1U). Meanwhile, the down expression of Upb1 further promoted the protein expression of renal tubular injury marker NGAL (Fig. 1V and 1W). In summary, these findings indicated that Upb1 knockdown exacerbated IR-induced AKI, but the specific mechanism was still unclear.

### Upb1 downregulation after AKI drives 3-ureidopropionic acid accumulation and nuclear entry in proximal tubular cells to exacerbate injury

To investigate the potential mechanism of Upb1 in AKI, we first examined its fundamental physiological functions and reviewed the pyrimidine metabolism pathway (Fig. 2A). Previous studies had shown that β-ureidopropionase deficiency due to Upb1 variants could lead to pathological accumulation of 3-Ureidopropionic acid (3-UPA) in body fluids 21, 22. Therefore, we focused on the accumulation of 3-UPA in the kidney. We then employed a liquid chromatography-tandem mass spectrometry (LC-MS/MS)-based targeted metabolomics approach, which revealed that 3-UPA accumulation in the kidney increased after IR (Fig. 2B), and the knock down of Upb1 further exacerbated this accumulation (Fig. 2C). The kidney and liver are the main sites of hydrolysis metabolism of 3-UPA due to their high abundance of Upb1 expression. However, our prior data indicated no alteration in hepatic Upb1 expression in IR-AKI mice (Fig. 1F), implying that the decrease of Upb1 renal expression following AKI primarily drives the accumulation of 3-UPA. Subsequently, we overexpressed Upb1 in TKPTs *in vitro*. Under H/R conditions, targeted metabolomics revealed reduced accumulation of 3-UPA with Upb1 overexpression compared to the Vehicle group (Fig. 2D).

To validate the role of 3-UPA within cells, we exposed TKPTs to FITC-labeled 3-UPA and observed that the functional concentration of 3-UPA (10 mM) was capable of entering the nucleus and colocalizing with the nuclear marker DAPI (Fig. 2E). In addition, 3-UPA induced a dose-dependent upregulation in the expression of inflammatory factors MCP-1 and TNF-α (Fig. 2F and 2G). Under both normoxic and hypoxic conditions, 3-UPA resulted in elevated expression of cleaved caspase-3 (Fig. 2J and 2K), a marker of apoptosis, and consequently induced increased cellular apoptosis (Fig. 2H and 2I). Interestingly, overexpression of Upb1 in TKPTs partially reversed the apoptosis induced by 3-UPA treatment *in vitro* (Fig. 2L).

### 3-UPA aggravates FA- and IR-triggered AKI, counteracted by Upb1 overexpression

To assess the role of 3-UPA in AKI, we administered 3-UPA (180 mg/kg, twice daily for two days, intraperitoneally) to FA-AKI mice, and found that 3-UPA significantly exacerbated FA-induced AKI, leading to further increases in Scr and BUN levels (Fig. 3A and 3B). The damage revealed by pathological PAS staining was more severe (Fig. 3C and 3D). The expression level of NGAL, a marker of renal tubular injury, was further upregulated following 3-UPA administration (Fig. 3E and 3F). We got the similar results in IR-induced AKI mice after 3-UPA injection (Fig. 3G-K).

Additionally, when we administered a higher working concentration (260 mg/kg, twice daily for 2 days, i.p.) to FA-AKI mice, it resulted in the death of 3 out of 10 mice. The surviving mice displayed a similar degree of renal injury exacerbation as previously documented, confirmed through histological and functional evaluations (Fig. S3). Interestingly, administering 3-UPA to non-AKI mice did not induce any detectable impairment in renal function (Fig. 3A-D), suggesting that 3-UPA may not exhibit inherent nephrotoxicity under normal physiological conditions. All the above results strongly suggested that 3-UPA may play a role in renal tubular injury, although the precise mechanism remains to be elucidated.

To further validate the role of Upb1 regulating 3-UPA during AKI, we injected Upb1 overexpression plasmids into the tail vein of FA-AKI mice that had been treated with 3-UPA (Fig. 3Q and 3R), following the previously described modeling protocol. Targeted metabolomics revealed that renal 3-UPA accumulation was significantly reduced in mice overexpressing Upb1 compared with the Vehicle group (Fig. 3L). The indicators of renal function, Scr and BUN, were also lower in the Upb1 overexpression group (Fig. 3M and 3N), a finding corroborated by PAS staining, which revealed a corresponding reduction in renal tubular injury (Fig. 3O and 3P). In addition, we observed a significant reduction in NGAL protein expression within the renal tissue of the Upb1 overexpression group (Fig. 3Q and 3R). These results suggested that Upb1 overexpression facilitated the catabolism of 3-UPA, thereby reducing its renal accumulation and ameliorating FA-induced acute kidney injury.

### Multi-omics analysis identifies 3-UPA as a potential regulator of fatty acid metabolism through DNMT1-mediated DNA methylation

To further investigate the mechanism of 3-UPA exacerbated renal tubular injury, we treated TKPTs with 3-UPA (10mM) for 24 hours *in vitro* and sent it for RNA-sequencing analysis. A total of 666 differential expressed genes (DEGs) from the gene expression RNA-seq data (Fig. 4A and 4B), including 175 up-regulated and 491 down-regulated, were identified as statistically significant (log fold change [log_2_FC] > 1.5). Kyoto Encyclopedia of Genes and Genomes (KEGG) pathway analysis revealed that these DEGs were predominantly enriched in the “DNA-templated transcription pathway”, while the remaining pathways were primarily associated with genetic modifications as well (Fig. 4C).

Subsequently, we verified the expression of three crucial DNA methyltransferases (DNMT1, DNMT3a, and DNMT3b) after 3-UPA treatment of TKPTs. Notably, the protein expression of DNMT1 exhibited an increase after 3-UPA treatment (Fig. 4D and 4E). Parallel to this, we assessed whole DNA methylation levels in TKPTs exposed to 3-UPA for 24 hours using a colorimetric assay. Our findings revealed a substantial elevation in overall DNA methylation levels within the cell samples (Fig. 4F). To delve deeper into genome-wide alterations in DNA methylation, we employed reduced representation bisulfite sequencing (RRBS), enabling us to pinpoint DNA methylation with single-base precision. A total of 2.5 billion and 4.9 billion of CpG sites were analyzed in the control and 3-UPA-treated TKPTs samples, respectively. We identified 3,836 differentially methylated regions (DMRs) between the two groups that showed notable (>±0.2) differences in methylation. These DMRs were associated with 2,446 genes, among which 448 genes had DMRs located in upstream promoter regulatory regions (Fig. 4G).

Since promoter regions are generally considered critical for transcriptional regulation, we further performed gene ontology (GO) and KEGG analyses on these 448 genes. A striking feature of the GO data was the prevalence of negative regulation across biological processes. The prominent role of lipid metabolic regulation, specifically the negative regulation of fatty acid oxidation, implies a hindrance to lipid breakdown, favoring processes like energy storage or membrane synthesis (Fig. 4H). The directed acyclic graph (DAG), serving as a graphical display of the GO enrichment analysis outcomes, emphasized the biological processes (BPs) significantly modulated by 3-UPA. The enrichment of terms such as “organic acid metabolic process” (GO:0006082), “lipid metabolic process” (GO:0006629), and “fatty acid metabolic process” (GO:0006631) indicates that 3-UPA has the potential to impact lipid and fatty acid metabolism, which are pivotal for maintaining energy homeostasis and cellular signaling (Fig. 4I). The KEGG pathway classification results similarly revealed that 3-UPA treatment had a considerable effect on metabolic signaling pathways (Fig. 4J). Based on the above results, we hypothesize that 3-UPA may affect fatty acid metabolism in renal tubular cells through DNMT1-mediated DNA methylation (Enlarged versions of all key RRBS results was shown in Fig. S4).

To further validate our above hypothesis, we first needed to determine whether 3-UPA affected cellular fatty acid metabolism. We treated TKPTs with 3-UPA for 24 hours and, through oil red staining, observed a significant increase in lipid droplet accumulation under hypoxia-reoxygenation injury conditions (Fig. 4K). In mice with AKI induced by FA and IR, an increase in lipid droplet accumulation was similarly noted on frozen kidney tissue sections following 3-UPA treatment (Fig. 4L-N). Subsequently, we performed oil red O staining on kidney tissues from AKI mice with either knockdown or overexpression of Upb1. Downregulating Upb1 resulted in renal accumulation of 3-UPA, accompanied by increased lipid droplet deposition as indicated by oil red O staining (Fig. S5A and 5B). Overexpression of Upb1 enhanced 3-UPA metabolism and reduced its renal accumulation, which corresponded to a decrease in lipid droplets observed via oil red O staining (Fig. S5E and 5F). These findings collectively indicated that 3-UPA served as a potential regulator of fatty acid metabolism in renal tubular cells.

### 3-UPA treatment causes mitochondrial dysfunction and is rescued partly by DNMT1 inhibition

Renal tubular epithelial cells primarily rely on fatty acid metabolism, particularly fatty acid β-oxidation (FAO), for energy production. FAO stands as a pivotal process in cellular energy metabolism, critically reliant on mitochondrial structure and function. Therefore, we employed the Seahorse XF Cell Mito Stress Test Kit to assess mitochondrial function in TKPTs following 3-UPA administration. The oxygen consumption rate (OCR) data indicated that after 24 hours of 3-UPA exposure, there was a notable decrease in basic respiration, ATP production and maximal respiration (Fig. 5A-D). Moreover, as the treatment duration extended to 48 hours, the aforementioned cellular parameters further declined, including spare respiratory capacity (Fig. 5A-E). After treating TKPTs with 3-UPA, we also observed a decrease in protein expression of respiratory chain complexes II, III, and V (Fig. 5G). Transmission electron microscopy (TEM) further disclosed that 3-UPA treatment led to excessive mitochondrial morphological damage in TKPTs (Fig. 5H). TEM also showed that compared with WT mice, mitochondria in the renal proximal tubules of Upb1 knockdown IR mice exhibited more severe structural abnormalities (Fig. S5D). Typically, injured mitochondria exhibit unstable membrane potential, thereby impacting the force driving fatty acids into the mitochondria. Consistent with this, the TMRM dye detection confirmed a reduction in mitochondrial membrane potential after 3-UPA exposure (Fig. 5F).

In addition, succinate dehydrogenase (SDH) is a key enzyme in mitochondrial energy metabolism, which is involved in the TCA cycle and electron transport chain (ETC), and is closely related to FAO and mitochondrial function. Our observations in kidney tissues from AKI mice induced by FA and IR revealed a decrease in SDH expression after 3-UPA administration (Fig. 5I and 5J). Subsequently, we performed SDH staining on kidney tissues from AKI mice following either Upb1 knockdown or overexpression. Downregulation of Upb1 resulted in renal accumulation of 3-UPA and a concurrent decrease in SDH expression (Fig. S5C). Conversely, Upb1 overexpression enhanced 3-UPA metabolism, reduced its renal accumulation, and was along with increased SDH staining (Fig. S5G).

Based on previous findings, we hypothesized that inhibiting DNMT1 might rescue 3-UPA-induced apoptosis in renal tubular epithelial cells. To test this, we used si-DNMT1 RNA to suppress the 3-UPA-induced upregulation of DNMT1 expression in TKPTs (Fig. 5K). We then observed that under H/R conditions, oil red O staining revealed a reduction in 3-UPA-induced lipid droplet accumulation following DNMT1 inhibition (Fig. 5L). After DNMT1 inhibition, the protein expression of respiratory chain complexes III and V, which were reduced under 3-UPA stimulation, partially recovered (Fig. 5M). Similarly, tubular epithelial cells apoptosis induced by 3-UPA also significantly decreased after DNMT1 inhibition (Fig. 5N).

### Meis1 acts as an upstream transcriptionally repressing regulator of Upb1 in AKI

Gene expression requires the mutual recognition and binding of transcription factors to promoters for initiation. To investigate the regulatory mechanism underlying the decline in Upb1 expression during renal tubular epithelial cell injury, we utilized the Genomatix (http://www.genomatix.de) and JASPAR (http://jaspar.genereg.net/) transcription factor analysis databases to predict potential transcription factors binding the Upb1 promoter region. This analysis revealed that myeloid viral integration site-1 (Meis1) possessed several binding sites within the Upb1 promoter. Subsequently, we performed experimental validation. ChIP assay in HEK-293T cells, guided by JASPAR predictions, identified three potential Meis1 binding sites at -711 bp, -441 bp, and -317 bp upstream of the human UPB1 transcription start site; all were confirmed to bind Meis1 (Fig. 6A). In mouse TKPTs, CUT&RUN assays validated significant Meis1 binding at -840 bp and +54 bp relative to the transcription start site (Fig. 6B), indicating that Meis1 directly interacts with the Upb1 promoter. Then we constructed a luciferase reporter containing the full-length mouse Upb1 promoter and performed dual-luciferase assays. Meis1 overexpression significantly suppressed Upb1 promoter activity (Fig. 6C).

To further validate the role of Meis1 in the pyrimidine metabolism pathway, we detected the expression of Upb1, DPYD and DPYS, three key enzymes of the 3-UPA metabolic pathway in TKPTs after transfection with Meis1 overexpression plasmid (Fig. 6D and S6A-B). The mRNA expression of Upb1 gene was significantly decreased (Fig. 6D), and the downregulation was mirrored in the protein expression of Upb1 following Meis1 overexpression (Fig. 6E).

Turan *et al*. 35 identified the key conserved amino acids in the Meis1 homeodomain that interact with target DNA, and developed two small-molecule Meis1 inhibitors accordingly. Both of these inhibitors can inhibit the transcriptional activity of Meis1 in a dose-dependent manner in HEK-293T cells. To further investigate the role of Meis1 on Upb1 in renal tubular epithelial cells, we synthesized a compound called MEISi-2 (MEISi). we treated human tubular epithelial cells with MEISi and assessed Upb1 expression. The results indicated that MEISi could enhance the protein expression of Upb1 in a dose-dependent manner (Fig. 6F). All these findings demonstrated that Meis1 could transcriptionally repress Upb1.

### Meis1 is up-regulated in the kidneys of AKI

To examine the association of Meis1 with AKI, we performed IHC staining with biopsied kidney tissues from 10 patients with AKI. Compared with the low levels in control human kidneys, significant upregulation of MEIS1 was observed in AKI patients' kidneys (Fig. 6G and 6H). Furthermore, analysis of the integrated optical density (IOD) correlation curve indicated a negative correlation between the expression of UPB1 (as shown in Fig. 1D) and MEIS1 in the kidneys of AKI patients (Fig. 6I).

Subsequently, we further verified the distribution of MEIS1 in renal tubules by detecting the fluorescence co-localization of MEIS1 and the tubule marker lotus tetragonolobus lectin (LTL) in kidney tissues of AKI patients (Fig. 6J). Then, we detected the Meis1 expression in FA-AKI model. We found that Meis1 expression increased significantly in the kidney at mRNA levels (Fig. 6K), with the protein level of expression being much greater in the nuclei compared to that in the cytoplasm in FA model (Fig. 6L).

### Tubule-specific Meis1 overexpression aggravates AKI and promoted cell apoptosis by upregulating 3-UPA accumulation

To evaluate the role of Meis1 in AKI, we generated proximal tubule Meis1 conditional knock-in (cKI) mice and verified the overexpression of Meis1 in tubular cells (Fig. S7). We then established the FA and IR models using Meis1-cKI and WT mice. Specific overexpression of Meis1 in renal tubule cells significantly aggravated the kidney injury. In the two AKI models, compared with WT mice, cKI mice showed a significant deterioration of renal function, with further increases in the levels of BUN (Fig. 7A and 7C) and Scr (Fig. 7B and 7D). PAS staining revealed significantly aggravated pathological damage, manifested as further dilation of renal tubules, disappearance of the brush border, shedding of renal tubular cells, and formation of casts (Fig. 7E and 7F). The tubular injury score was significantly elevated (Fig. 7G and 7H). Meanwhile, the TUNEL results showed that the overexpression of Meis1 significantly promoted the apoptosis of renal tubular cells in two AKI models (Fig. 7I-L). Furthermore, we overexpressed Meis1 in TKPTs *in vitro* (Fig. S8A), and observed a significant increase in apoptosis under H/R condition compared with vehicle group (Fig. S8B).

To further investigate whether Meis1 regulates pyrimidine catabolism via suppression of Upb1, we first performed a LC-MS/MS-based non-targeted metabolomics in the kidneys of transgenic mice after IR. Compared with the WT group, there were 13 metabolites with statistically significant differences in the Meis1 cKI group under positive ion (POS) mode (Fig. 7M and 7N). Surprisingly, the most significantly upregulated metabolite was 3-UPA, which is involved in pyrimidine metabolism (Fig. 7O). Further verification by targeted metabolomics revealed that knock-in Meis1 exacerbated 3-UPA accumulation in the kidney increased after IR (Fig. 7P). Therefore, the increased 3-UPA in the kidney indirectly reflects more severe renal tubular injury, which is consistent with our previous observations.

Additionally, renal tubules from Meis1-cKI and WT mice underwent RNA-sequencing analysis. The analysis identified 3060 DEGs from the gene expression RNA-seq data, with 1591 genes up-regulated and 1469 genes down-regulated, all statistically significant (Fig. S9A). GO enrichment analysis indicated that these DEGs were predominantly enriched in the “oxidoreductase activity” (Fig. S9B), and the pathways enrichment was also related to “Oxidative phosphorylation” and “Metabolic pathways” (Fig. S9C). In both IR and FA induced AKI models using Meis1-cKI and WT mice, we observed an elevation in lipid droplet accumulation in the kidney tissues of Meis1-cKI mice (Fig. S9D and S9E). All the aforementioned phenotypes maybe a result of Meis1 upregulated the accumulation of 3-UPA by transcriptionally repressing Upb1.

### The Meis1 inhibitor MEISi alleviates AKI induced by FA, IR and suppressed cell apoptosis

As described previously, we synthesized the compound MEISi to investigate the effects of inhibiting Meis1 transcriptional activity in AKI. After optimizing the conditions in preliminary experiments, we treated mice with MEISi at 0.4 mg/kg/day via i.p. injection 24h before FA delivery or IR surgery. Then the mice were treated daily for 2 consecutive days and sacrificed.

In FA model, compared with the con+FA group, the MEISi+FA group exhibited a decrease in Scr (Fig. 8A), attenuation of renal tubular dilation and protein casts, which was associated with a lower tubular injury score (Fig. 8B and 8C). qPCR showed that MEISi inhibited the expression of KIM-1, NGAL and MCP-1(Fig. 8D-F). The apoptosis of renal tubular cells also reduced as shown by TUNEL assay (Fig. 8G and 8H). The determination of the concentrations of serum alanine aminotransferase (ALT), aspartate aminotransferase (AST), creatine kinase-MB (CK-MB) and lactate dehydrogenase (LDH) indicated that therapeutic doses of MEISi had no obvious hepatic, heart, and systemic toxicity (Fig. S10A-D). To further validate the role of MEISi in the pyrimidine metabolism pathway, we detected the expression of Upb1, DPYD and DPYS in C57BL/6J mice kidneys treated with MEISi (Fig. S11A-S11C). The mRNA expression of Upb1 but not DPYD and DPYS was significantly increased (Fig. S11A).

In IR mice, similar to the FA model, MEISi also improved the decline in renal function (Fig. 8I) and renal tubular injury (Fig. 8J and 8K) induced by IR. The protein expression of renal tubular injury markers KIM-1 and NGAL partially decreased after MEISi treatment in IR model (Fig. 8L and 8M). Oil red O staining further revealed a reduction in renal lipid droplet accumulation following MEISi treatment after IR (Fig. 8N and 8O). Together, these data reveal that the Meis1 inhibitor MEISi alleviated AKI induced by FA and IR.

Besides, in cultured TKPTs, the Meis1 inhibitor MEISi demonstrated the capacity to suppress the increase of apoptosis triggered by hypoxia-reoxygenation (Fig. S8C).

## Discussion

AKI represents a prevalent clinical syndrome linked to elevated morbidity and mortality rate 36. Pinpointing possible therapeutic targets capable of influencing AKI holds utmost importance in formulating innovative treatment strategies to combat this condition 37. The kidneys, especially proximal tubules, rely on high energy metabolism to sustain their function. Inhibition of FAO during AKI leads to intracellular lipid droplet accumulation, diminished ATP production, and cell apoptosis, which constitute central metabolic mechanisms underlying disease progression 11, 38. The present study marks the first demonstration that 3-UPA, an intermediate in pyrimidine catabolism, plays a potential regulatory role in fatty acid metabolism in proximal tubular epithelial cells during AKI. Our systematic analysis of multi-omics data, combined with functional assays, revealed an unexpected regulatory axis from Meis1 to 3-UPA mediated through transcriptional repression of Upb1, and suggested that the Meis1/Upb1/3-UPA axis may aggravate AKI by inducing fatty acid oxidation disorders and mitochondrial dysfunction, possibly via DNMT1-mediated DNA methylation.

Proximal tubular epithelial cells represent the main cell population affected in AKI. Through analysis of a single-cell sequencing database, we discovered that Upb1 is primarily expressed in renal proximal tubular epithelial cells. Furthermore, both *in vivo* and *in vitro* investigations indicate a sharply decrease in Upb1 expression in TKPTs following injury. Upb1 encodes β-ureidopropionase, a key enzyme catalyzes the last step in the pyrimidine degradation pathway 39. Extensive experimental data demonstrate that bi-allelic damaging variants in Upb1 lead to a deficiency in β-ureidopropionase activity. Reports have linked these Upb1 variants to a range of neurodevelopmental phenotypes, such as intellectual disability, seizures, and autism 40-42. Libin Zhang *et al*. showed that low UPB1 is correlated with a worse prognosis of Lung adenocarcinoma (LUAD) and may be a valuable prognostic indicator for LUAD 43. Thus, the link between UPB1 deficiency and clinical phenotypes remains unclear, highlighting a long-overlooked but critical role for Upb1 in disease progression. Here, we demonstrate for the first time that Upb1 confers protection in acute kidney injury.

A recent study has found that pyrimidines, but not purines, maintain pyruvate oxidation and the TCA cycle by regulating pyruvate dehydrogenase activity, thereby influencing adipocyte differentiation and lipid production. Insufficient pyrimidine synthesis impairs the ability of preadipocytes to differentiate into mature adipocytes 16. This finding provided direct evidence that pyrimidine synthesis participates in fatty acid metabolism. However, whether pyrimidine catabolism is involved in fatty acid metabolism remains unclear. In our study, downregulation of Upb1 in AKI kidneys resulted in local accumulation of 3-UPA, a key intermediate in pyrimidine catabolism. Research on 3-UPA is limited, only one basic study indicating that 3-UPA can trigger an increase in mitochondrial reactive oxygen species production and a delayed rise in Ca^2+^ concentration in chicken neuronal cells, ultimately leading to neurodegeneration 23, hinting at potential toxic effects. Through integrated multi-omics analyses combined with biochemical and pathological experiments, we found that 3-UPA may disrupt fatty acid metabolism and impair mitochondrial function via DNMT1-mediated DNA methylation, leading to increased apoptosis in TKPTs. Our results reveal for the first time that 3-UPA serves as a critical hub linking pyrimidine catabolism and fatty acid metabolism.

Interestingly, our study showed that administering 3-UPA to non-AKI mice did not induce any detectable impairment in renal function, suggesting that 3-UPA may not exhibit inherent nephrotoxicity under normal physiological conditions. The observation echoes the “Triple Whammy acute kidney injury” phenomenon 44, wherein pre-existing renal impairment can exacerbate the nephrotoxic effects of certain agents, possibly due to metabolite accumulation or the amplification of specific renal mechanisms.

Considering that Upb1 shows a significant decrease at both the mRNA and protein levels during AKI, it is speculated that transcriptional regulation may be involved. Transcription factor analysis revealed that Meis1 could transcriptionally repress Upb1. Meis1 was first identified as a critical regulator of myeloid leukemia development in BXH-2 mice 45. Subsequent studies have revealed that Meis1 plays a pivotal role in controlling cell fate by modulating key processes such as apoptosis, cell cycle progression, and cellular metabolism, mainly in cancer 46. Kocabas F *et al*. reported that Meis1 deletion in hematopoietic stem cells downregulates both Hif-1α and Hif-2α, triggering a shift toward mitochondrial metabolism, elevated reactive oxygen species production, and consequent cell apoptosis 25. Here we demonstrated that Meis1 overexpression exacerbated FA- and IR-induced AKI and promoted tubular epithelial cell apoptosis. Conversely, the Meis1 inhibitor MEISi attenuated AKI in both models. Furthermore, untargeted metabolomics revealed that 3-UPA accumulated following Meis1 overexpression, further confirming that Meis1 promotes 3-UPA accumulation in the kidney by repressing Upb1, thereby disrupting the fatty acid metabolism. These results uncover a novel metabolic function of Meis1 in modulating pyrimidine catabolism, which in turn governs fatty acid metabolism.

Both our previous 27 and present studies and those conducted by Chang-Panesso *et al*. 47 have consistently shown an increase in the expression level of Meis1 in injured kidneys. However, discrepancies exist regarding its precise localization. In this study, IHC revealed strong Meis1 expression in renal tubules of AKI patients, and immunofluorescence confirmed colocalization with the tubular marker LTL. In contrast, Chang-Panesso *et al*. reported predominant Meis1 localization in interstitial cells in mouse models and human kidneys, likely due to differences in sampling timing and patient populations.

In addition, our study has certain limitations. The precise mechanisms by which 3-UPA regulates DNMT1 expression and participates in DNA methylation remain to be further investigated. Moreover, which fatty acid metabolism-related genes are specifically methylated by DNMT1 also requires additional experimental validation. Notably, our findings suggest that 3-UPA might serve as a novel biomarker for disease. Regarding detection feasibility, 3-UPA is a terminal metabolite of the pyrimidine degradation pathway with good aqueous solubility. It can be reliably quantified in both urine and plasma using established LC-MS/MS or gas chromatography-mass spectrometry (GC-MS) approaches 48. Among these biofluids, urine offers a non-invasive sampling advantage that is particularly conducive to large-scale clinical screening. Nevertheless, the present findings are correlative, and well-designed prospective cohorts are required to validate the sensitivity, specificity, and predictive value of 3-UPA alone or in combination with other biomarkers before clinical translation can be realized. Moreover, the current study is based on cell lines and animal models, and extending these findings to human-derived organoids and drug development pipelines could be important future direction.

Collectively, our data provide a novel metabolic perspective on AKI pathogenesis. Meis1 represses Upb1 transcriptionally, resulting in 3-UPA accumulation that exacerbates AKI, likely through DNMT1-mediated DNA methylation, which disrupts fatty acid oxidation and mitochondrial function. Therapeutic targeting of Meis1 or activation of the Upb1 pathway could offer a novel approach to restore renal mitochondrial homeostasis and mitigate AKI progression.

## Supplementary Material

Supplementary figures and tables.

## Figures and Tables

**Figure 1:**
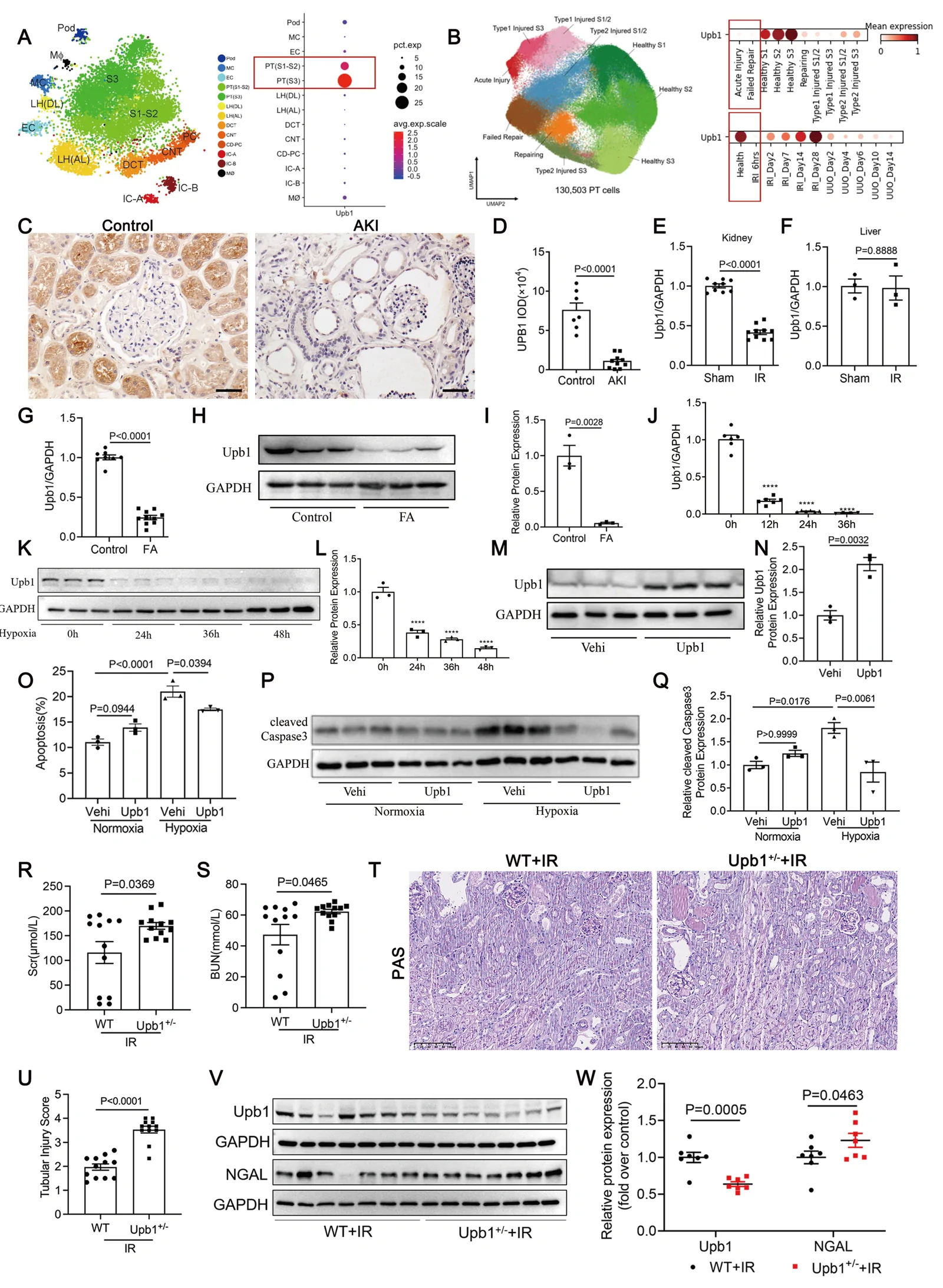
** UPB1 is down-regulated in the kidneys of AKI patients and mice and global knockdown of Upb1 aggravates AKI.** (**A** and** B**) The expression of Upb1 in the Kidney Interactive Transcriptomics database. (**C**) Immunohistochemical analysis of UPB1 expression in AKI children (n=10). N=7 in Normal group. Scale bar: 50μm. (**D**) Immunohistochemical semi-quantitative IOD analysis of UPB1. (**E** and** G**) qRT-PCR analysis of Upb1 in kidney after IR or FA model (n=8-10). (**F**) qRT-PCR analysis of Upb1 in liver after IR (n=3). (**H** and** I**) Western blot and densitometric analysis for the expression of Upb1 in FA model (n=3). (**J, K** and** L**) qRT-PCR and Western blot analysis for the expression of Upb1 in TKPTs followed by hypoxia (1%O_2_) culture for different time and reoxygenation for 2h (n=3 or 6). (**M** and** N**) Western blot and densitometric analysis for the expression of Upb1 after transfection with Upb1 plasmid for 48h (n=3). (**O**) Quantification of apoptosis of TKPTs after transfection with Upb1 plasmid followed by H/R incubation (n=3). (**P** and** Q**) Western blot and densitometric analysis for the expression of cleaved-caspase3 after transfection with Upb1 plasmid followed by H/R incubation (n=3). (**R** and **S**) Analysis of Scr and BUN of Upb1^ +/-^ mice and WT mice in IR-AKI model (n=12). (**T**) Representative images for PAS staining of Upb1^ +/-^ mice and WT mice after IR. Scale bar: 100μm. (**U**) Quantification of tubular injury score according to PAS staining of Upb1^ +/-^ mice and WT mice after IR (n=12). (**V** and **W**) Western blot and densitometric analysis for the protein expression of Upb1 and NGAL in Upb1^ +/-^ mice and WT mice in IR-AKI model (n=7). Data are presented as mean ± SEM. Data were statistically analyzed using unpaired Two-tailed Student's t-test (**D, E, G, I, J, L, N, R, S, U** and** W**) or One-way ANOVA (**O** and **Q**). The P-values were shown in the figures. **** mean p < 0.0001 *vs*. 0h.

**Figure 2:**
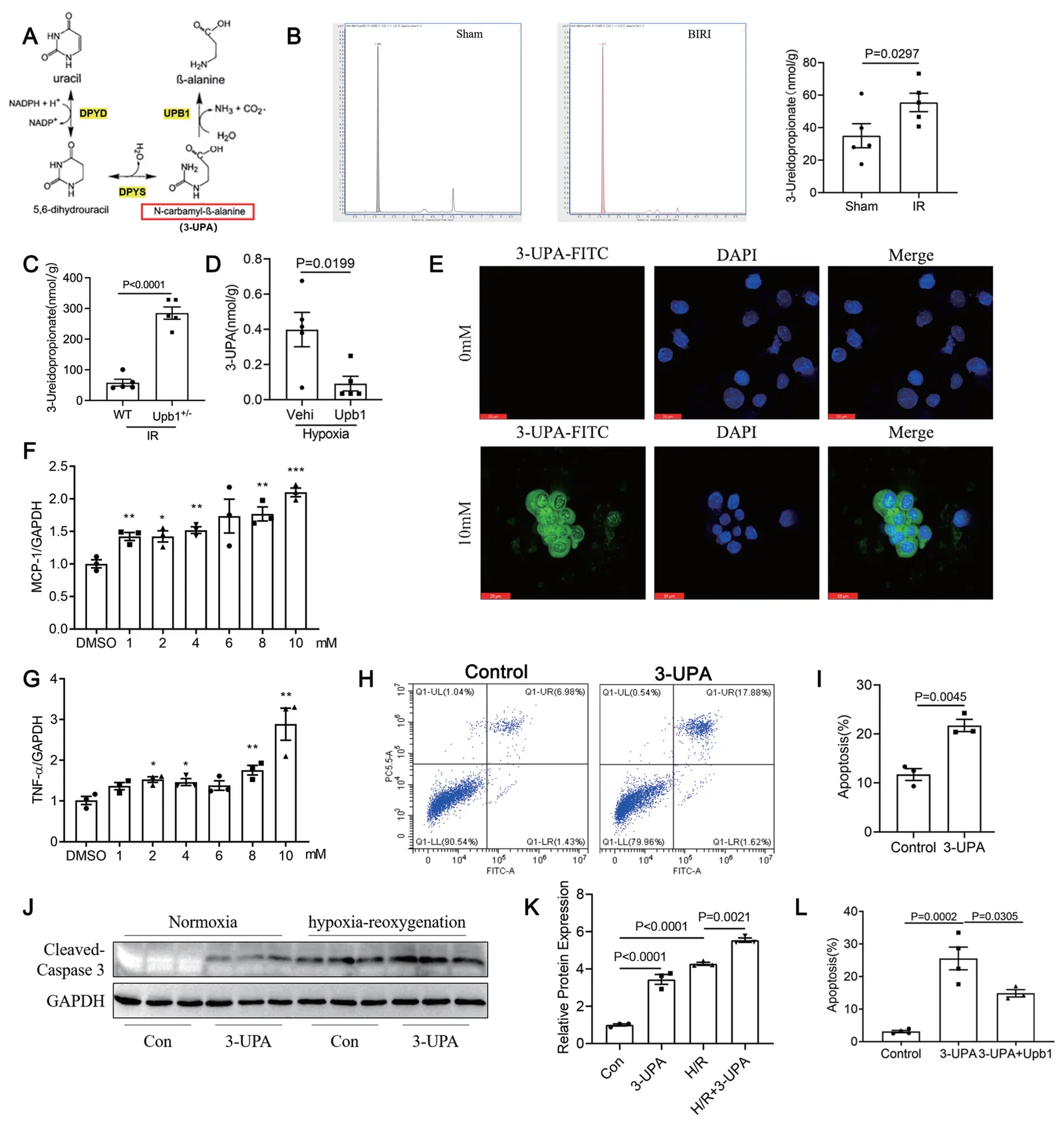
** 3-UPA is regulated by renal Upb1 and entered into nuclear of the TKPTs, promoting inflammation and apoptosis.** (**A**) Diagram of pyrimidine metabolism. (**B**) Targeted metabolomic analysis of renal 3-UPA by LC-MS/MS in IR model (n=5). (**C**) Targeted metabolomic analysis of renal 3-UPA by LC-MS/MS in Upb1^ +/-^ mice and WT mice after IR (n=5). (**D**) Targeted metabolomic analysis of 3-UPA by LC-MS/MS in TKPTs exposed to Upb1 overexpression plasmid followed by hypoxia (1%O_2_) culture for 24h and reoxygenation for 2h (n=5). (**E**) Representative fluorescence diagrams of the location of 3-UPA in TKPTs after 24h stimulation with 3-UPA-FITC (n=3). Scale bar: 20μm. (**F** and **G**) qRT-PCR analysis of MCP-1 and TNF-α in TKPTs exposed to 3-UPA at different concentrations for 24h (n=3). (**H** and **I**) Flow cytometry analysis using Annexin V-FITC/PI staining and apoptosis quantification of TKPTs after administration of high concentration 3-UPA (10mM) for 24h (n=3). (**J** and **K**) Western blot and densitometric analysis for the expression of cleaved-caspase3 after 3-UPA (1mM) treatment under normal oxygen or hypoxia-reoxygenation condition for 24h (n=3). (**L**) Apoptosis quantification of TKPTs exposed to 3-UPA (10mM) after overexpressing Upb1 for 24h (n=3). Data are presented as mean ± SEM. Data were statistically analyzed using unpaired Two-tailed Student's t-test (**B**, **C**,** D**,** F**,** G**,** I**,** J** and** K**) or One-way ANOVA (**L**). The P-values were shown in the figures. *, **, *** and **** mean p < 0.05, p < 0.01, p < 0.001 and p < 0.0001 *vs.* DMSO group, respectively.

**Figure 3:**
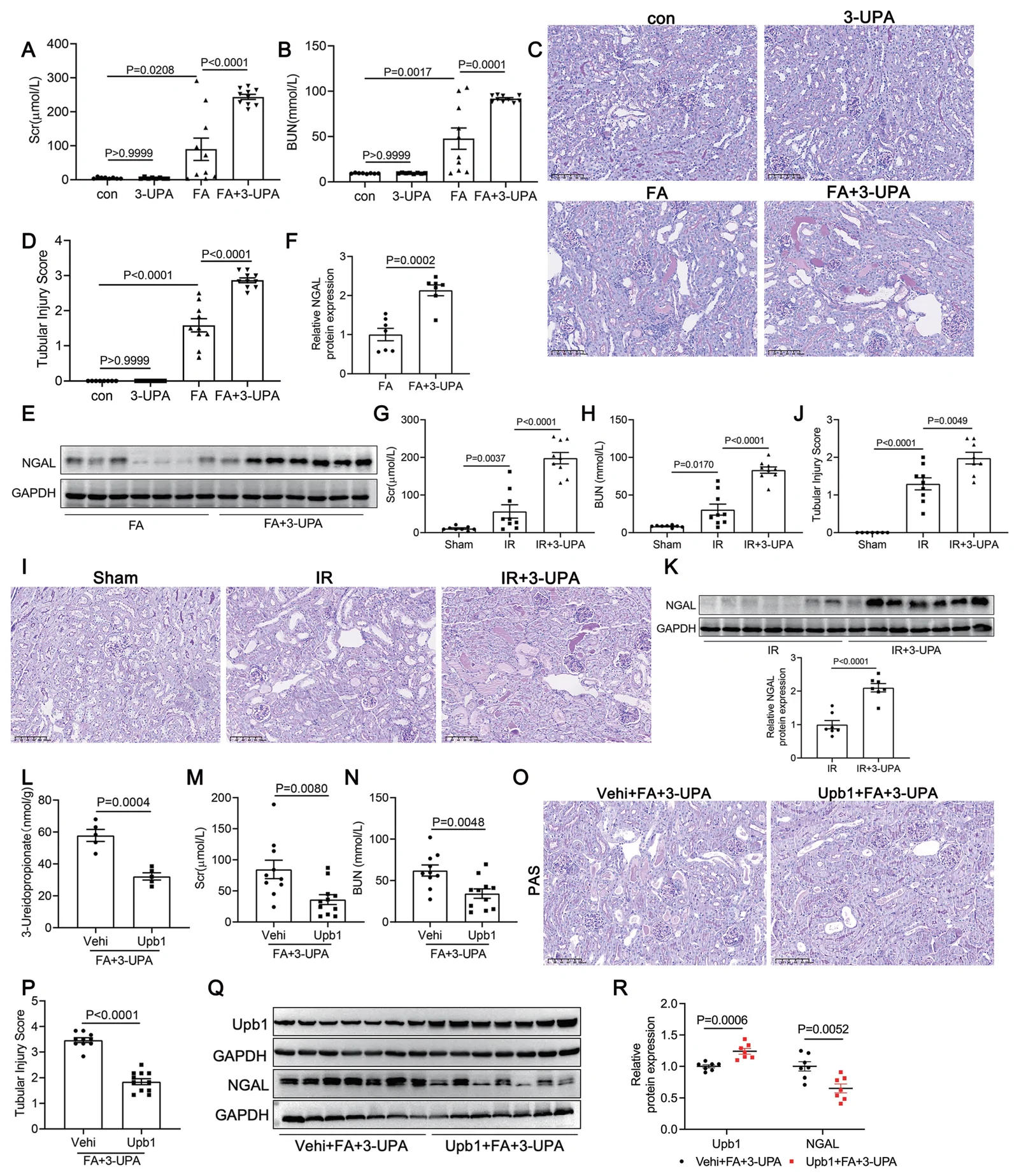
** 3-UPA aggravates FA and IR-induced AKI, whereas Upb1 overexpression mitigates renal damage.** (**A** and **B**) Analysis of Scr and BUN after FA model in WT mice treated with 3-UPA (n=8 in con or 3-UPA group, n=10 in FA or FA+3-UPA group). (**C**) Representative images for PAS staining of FA-induced AKI mice kidneys followed by 3-UPA treatment. Scale bar: 100μm. (**D**) Quantification of tubular injury score according to PAS staining of FA model in mice treated with 3-UPA administration (n=8 in con or 3-UPA group, n=10 in FA or FA+3-UPA group). (**E** and **F**) Western blot and densitometric analysis for the expression of NGAL in FA-induced AKI mice kidneys (n=7). (**G** and **H**) Analysis of Scr and BUN after IR model in WT mice treated with 3-UPA (n=8 in Sham group, n=9 in IR or IR+3-UPA group). (**I**) Representative images for PAS staining of IR-induced AKI mice kidneys followed by 3-UPA treatment. Scale bar: 100μm. (**J**) Quantification of tubular injury score according to PAS staining of IR model in mice (n=7 in Sham group, n=9 in IR group and n=8 in IR+3-UPA group). (**K**) Western blot and densitometric analysis for the expression of NGAL in IR-induced AKI mice kidneys treated with 3-UPA administration (n=7). (**L**) Targeted metabolomics analysis of renal 3-UPA in FA-AKI mice treated with 3-UPA after injecting an Upb1 overexpression plasmid into the tail vein (n=5). (**M** and **N**) Analysis of Scr and BUN in Upb1 overexpressed mice treated with 3-UPA after FA model (n=10 in Vehicle group and n=11 in Upb1 group). (**O**) Representative images for PAS staining of Upb1 overexpressed mice followed by 3-UPA treatment after FA model. Scale bar: 100μm. (**P**) Quantification of tubular injury score according to PAS staining of Upb1 overexpressed mice followed by 3-UPA treatment after FA model (n=10 in Vehicle group and n=11 in Upb1 group). (**Q** and **R**) Western blot and densitometric analysis for the protein expression of Upb1 and NGAL in Upb1 overexpressed mice followed by 3-UPA treatment after FA model (n=7). Data are presented as mean ± SEM. Data were statistically analyzed using unpaired Two-tailed Student's t-test (**F**, **K, L, M, N, P** and **R**) or One-way ANOVA (**A**, **B**, **D**, **G**, **H** and **J**). The P-values were shown in the figures.

**Figure 4:**
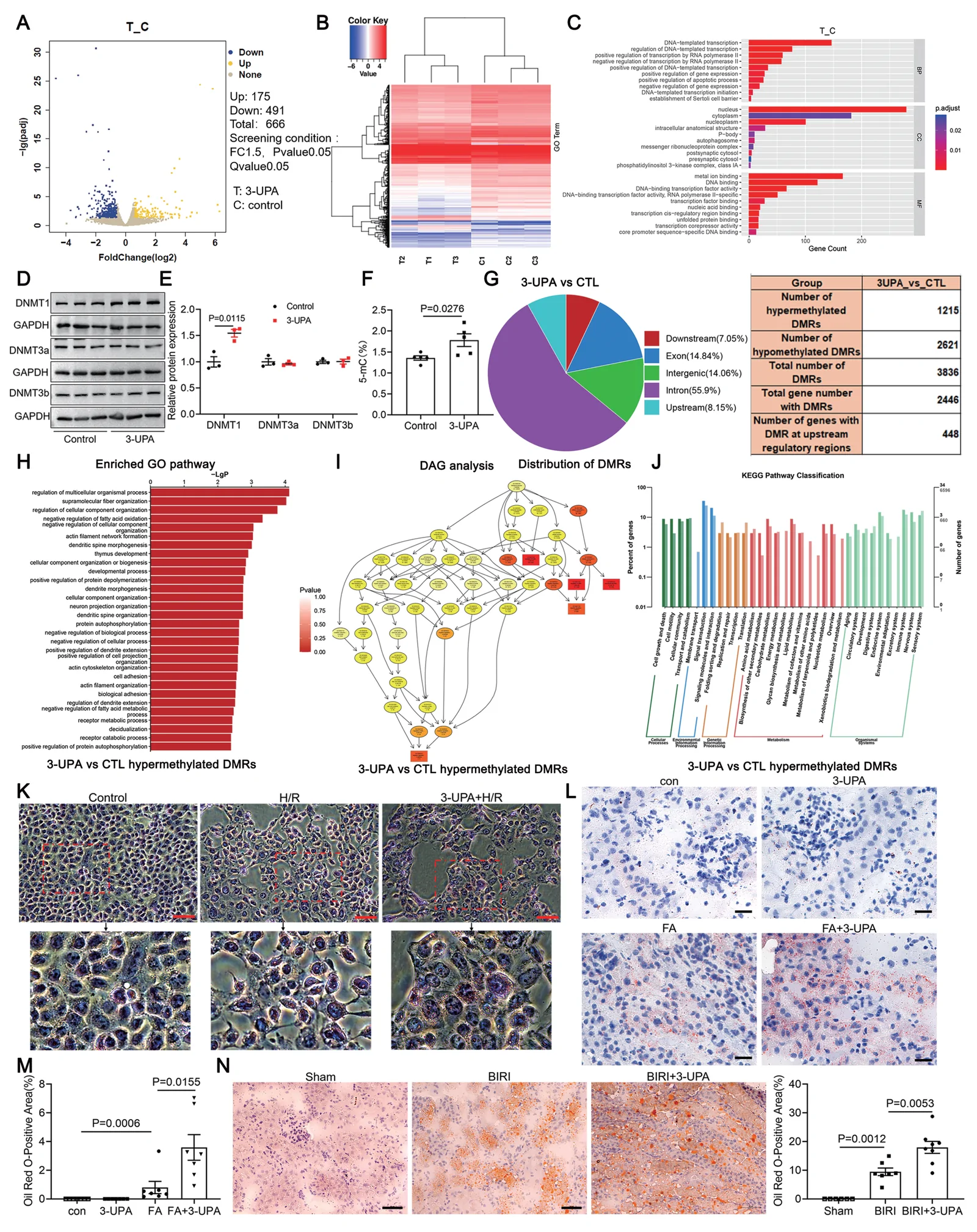
** 3-UPA treatment disruptes fatty acid oxidation through DNMT1-mediated DNA methylation based on multi-omics pathway enrichment analysis.** (**A** and **B**) Volcano-plot and Heatmap of differential expressed genes (DEGs) from the RNA-sequencing analysis of TKPTs with 3-UPA (10mM) stimulation for 24 hours (n=3). (**C**) Bar graph of GO enrichment analysis results of DEGs from the RNA-seq data in the BP, CC and MF terms. (**D** and **E**) Western blot and densitometric analysis for the expression of DNMT1, DNMT3a and DNMT3b in TKPTs after administration of high concentration 3-UPA (10mM) for 24h (n=3). Two-tailed Student's t-test. The P-value was shown in the figure. (**F**) Quantification of the whole DNA methylation levels in TKPTs exposed to 3-UPA (10mM) for 24 hours using a colorimetric assay (n=5). Two-tailed Student's t-test. The P-value was shown in the figure. (**G**) Location and numbers of differentially methylated regions (DMRs) from the RRBS analysis of TKPTs treated with 3-UPA (10mM) for 24 hours (n=3). (**H**) GO enrichment pathway analysis of hypermethylated DMRs in the 3-UPA group *vs.* control group. (**I**) The directed acyclic graph (DAG), serving as a graphical display of the GO enrichment analysis outcomes, emphasized the BPs significantly modulated by 3-UPA. (**J**) KEGG pathway classification results of hypermethylated DMRs in the 3-UPA group *vs.* control group. (**K**) Representative images for Oil Red O staining of TKPTs after 3-UPA (10mM) pretreatment for 0.5h followed by hypoxia (1%O_2_) culture for 24h and reoxygenation for 2h (n=3). Scale bar: 50μm. (**L** and** M**) Representative images and quantification for Oil Red O staining of kidney tissues of FA-induced AKI models treated with 3-UPA (n=7 in each group). Scale bar: 50μm. One-way ANOVA. The P-values were shown in the figure. (**N**) Representative images and quantification of Oil Red O staining positive area of kidney tissues in IR- induced AKI model treated with 3-UPA (n=6 in Sham group, n=7 in IR group and n=8 in IR+3-UPA group). Scale bar: 50μm. One-way ANOVA. The P-values were shown in the figure.

**Figure 5:**
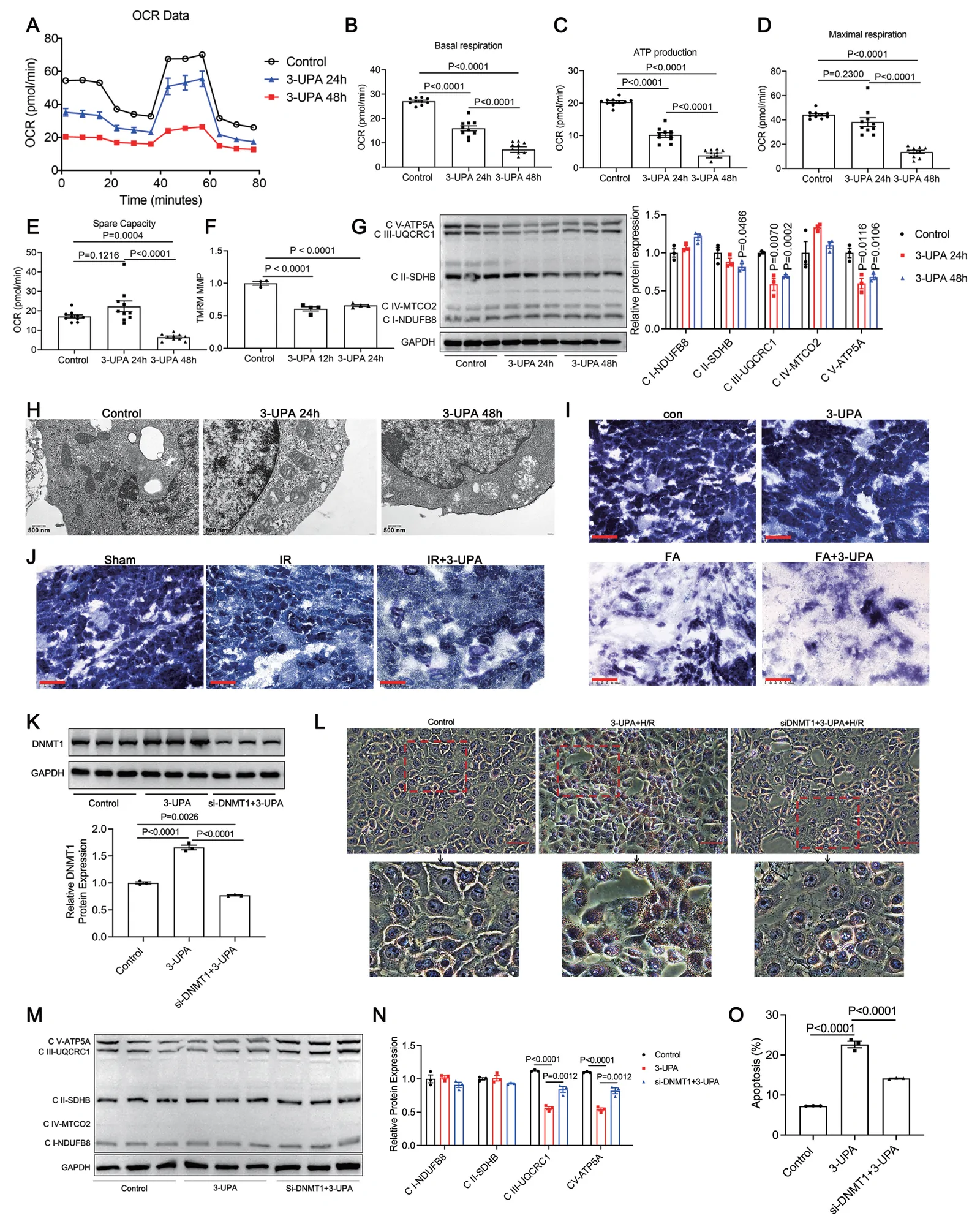
**3-UPA treatment causes mitochondrial dysfunction and is rescued partly by DNMT1 inhibition.** (**A**-**E**) Seahorse XF cell Mito stress test assay in the cultured TKPTs treated with 3-UPA (10mM) for 24 h or 48h. Quantification of oxygen consumption rate (OCR) for basal respiration, ATP production, maximal respiration and spare capacity (n=10). (**F**) Quantification of the mitochondrial membrane potential (MMP) using flow cytometry after TMRM staining in TKPTs exposed to 3-UPA (10mM) for 12 hours or 24 hours (n=4). (**G**) Western blot and densitometric analysis for the expression of respiratory chain proteins (subunits of complexes I, II, III, IV and Ⅴ) located in inner mitochondrial membrane of TKPTs after administration of 3-UPA (10mM) for 24h or 48h (n=3). (**H**) Representative transmission electron microscopy (TEM) images for mitochondria in the TKPTs exposed to 3-UPA (10mM) for 24h or 48h (n=3). Scale bar: 500nm. (**I** and **J**) Representative images for SDH staining of kidney tissues from AKI mice induced by FA and IR after 3-UPA administration. Scale bar: 100μm. (**K**) Western blot and densitometric analysis for the protein expression of DNMT1 in TKPTs exposed to 3-UPA (10mM) for 24 hours after treatment with si-DNMT1 RNA (80nM) (n=3). (**L**) Representative images for Oil Red O staining of TKPTs treated with si-DNMT1 RNA (80nM) for 24h and exposed to 3-UPA (10mM) pretreatment for 0.5h followed by hypoxia (1%O_2_) culture for 24h and reoxygenation for 2h (n=3). Scale bar: 50μm. (**M** and **N**) Western blot and densitometric analysis for the expression of respiratory chain proteins of TKPTs exposed to 3-UPA (10mM) for 24 hours after treatment with si-DNMT1 RNA (80nM) (n=3). (**O**) Quantification for the flow cytometry analysis after Annexin V-FITC/PI staining (n=3). TKPTs were transfected with si-DNMT1 RNA (80nM) for 24h before 3-UPA treatment (10mM). Data are presented as mean ± SEM. Data were statistically analyzed using One-way ANOVA (**B**, **C**, **D**, **E**, **F, G, K, N** and **O**). The P-values were shown in the figures.

**Figure 6:**
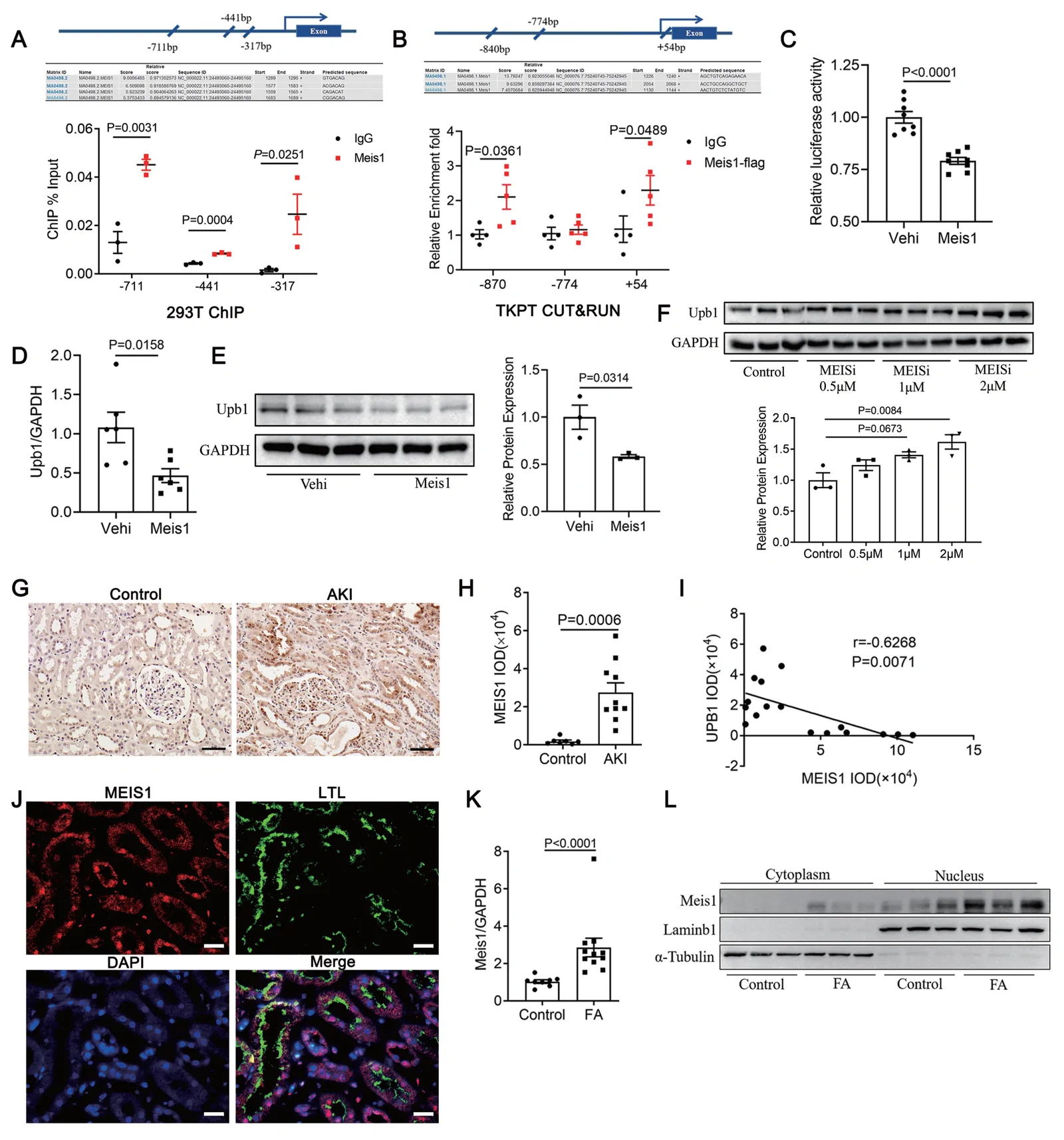
** Meis1 transcriptionally represses Upb1 and is up-regulated in the kidneys of AKI.** (**A** and** B**) Prediction of binding sites between Meis1 and Upb1 promoter. ChIP and CUT&RUN analysis of Meis1 at the regions of the Upb1 promoter (n=3). (**C**) Luciferase assay of TKPTs transfected with the pGL4.19-Upb1 promoter reporters with Meis1 plasmids (n=8). (**D**) qRT-PCR analysis of Upb1 in TKPTs after transfection with Meis1 overexpression plasmid for 24h (n=6). (**E**) Western blot and densitometric analysis for the expression of Upb1 after transfection with Meis1 plasmid for 48h (n=3). (**F**) Western blot and densitometric analysis for the expression of Upb1 after stimulation with different concentrations of MEISi in TKPTs (n=3). (**G**) Immunohistochemical analysis of MEIS1 expression in AKI children (n=10). N=7 in Normal group. Scale bar: 50μm. (**H**) Immunohistochemical semi-quantitative IOD analysis of MEIS1. (**I**) Pearson correlation analysis of MEIS1 and UPB1 in AKI patients (n=10). (**J**) Immunofluorescence co-localization analysis of MEIS1 and LTL in human kidney tissue. Scale bar: 20μm. (**K**) qRT-PCR analysis of Meis1 in FA-AKI model (n=8 in Control group, n=11 in FA group). (**L**) Western blot analysis of renal Meis1 in cytoplasm and nucleus in FA model. Lamin B1 and α-Tubulin was used as loading control (n=3). Data are presented as mean ± SEM. Data were statistically analyzed using unpaired two-tailed Student's t-test (**A**,** B**,** C**,** D**,** E**,** H** and **K**), One-way ANOVA (**F**) or Pearson correlation analysis (**I**). The P-values were shown in the figures.

**Figure 7:**
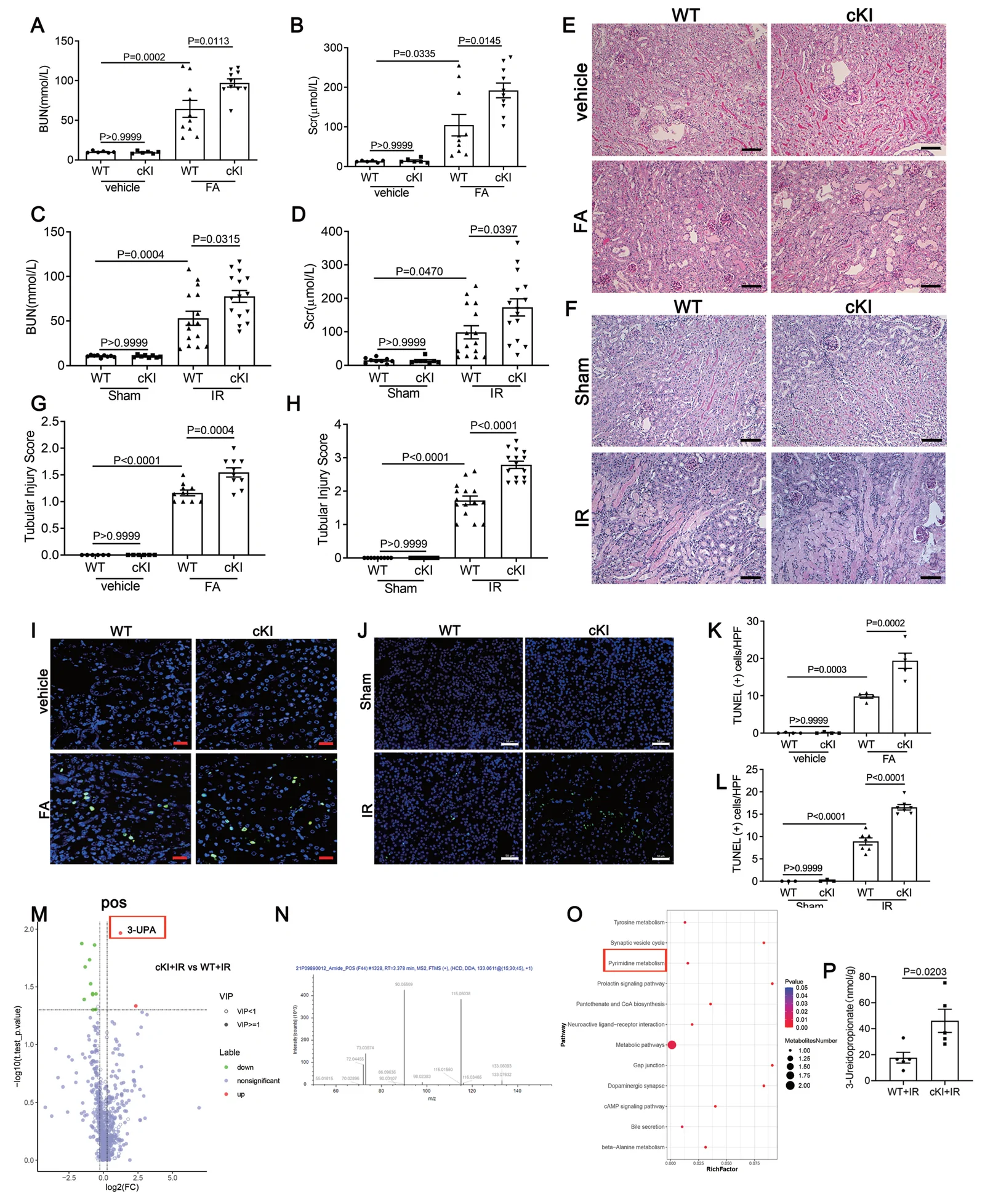
** Tubule-specific Meis1 overexpression aggravates AKI and promoted cell apoptosis by upregulating 3-UPA accumulation.** (**A** and** B**) Analysis of BUN and Scr after FA model in WT and Meis1 cKI mice. (**C** and** D**) Analysis of BUN and Scr after IR model in WT and Meis1 cKI mice. (**E** and** F**) Representative images for PAS staining of FA- and IR-induced AKI mice kidneys. Scale bar: 100μm. (**G** and** H**) Quantification of tubular injury score according to PAS staining of FA and IR model in WT and Meis1 cKI mice. In FA model, n=6 in Vehicle group, n=10 in FA group. In IR model, we combined the results of two independent experiments (n=9 in WT+Sham group, n=8 in cKI+Sham group, n=15 in WT+IR or cKI+IR group). (**I** and **J**) Representative images for TUNEL staining of FA- and IR-induced AKI mice kidneys. Scale bar: 50μm. (**K** and** L**) Quantification of tubular injury score according to TUNEL staining of FA (n=4) and IR (n=3 or 7) models in WT and Meis1 cKI mice. (**M**) Metabolite identification and quantitation in LC-MS/MS-based non-targeted metabolomics approach under positive ion (POS) mode to analyze the metabolites in the kidneys after IR in proximal tubule Meis1 cKI mice (log fold change [log_2_FC] ≥2, n=6 in each group). 3-UPA is circled in the red box. (**N**)The MS/MS spectrum of 3-UPA. (**O**) KEGG enrichment bubble chart of metabolic pathway analysis: the red box highlights pyrimidine metabolic pathway. (**P**) Targeted metabolomic validation of renal 3-UPA by LC-MS/MS after IR model in WT and Meis1 cKI mice (n=5). Data are presented as mean ± SEM. Data were statistically analyzed using unpaired two-tailed Student's t-test (**P**) or One-way ANOVA (**A**, **B**, **C**, **D**,** G**, **H**, **K** and **L**). The P-values were shown in the figures.

**Figure 8:**
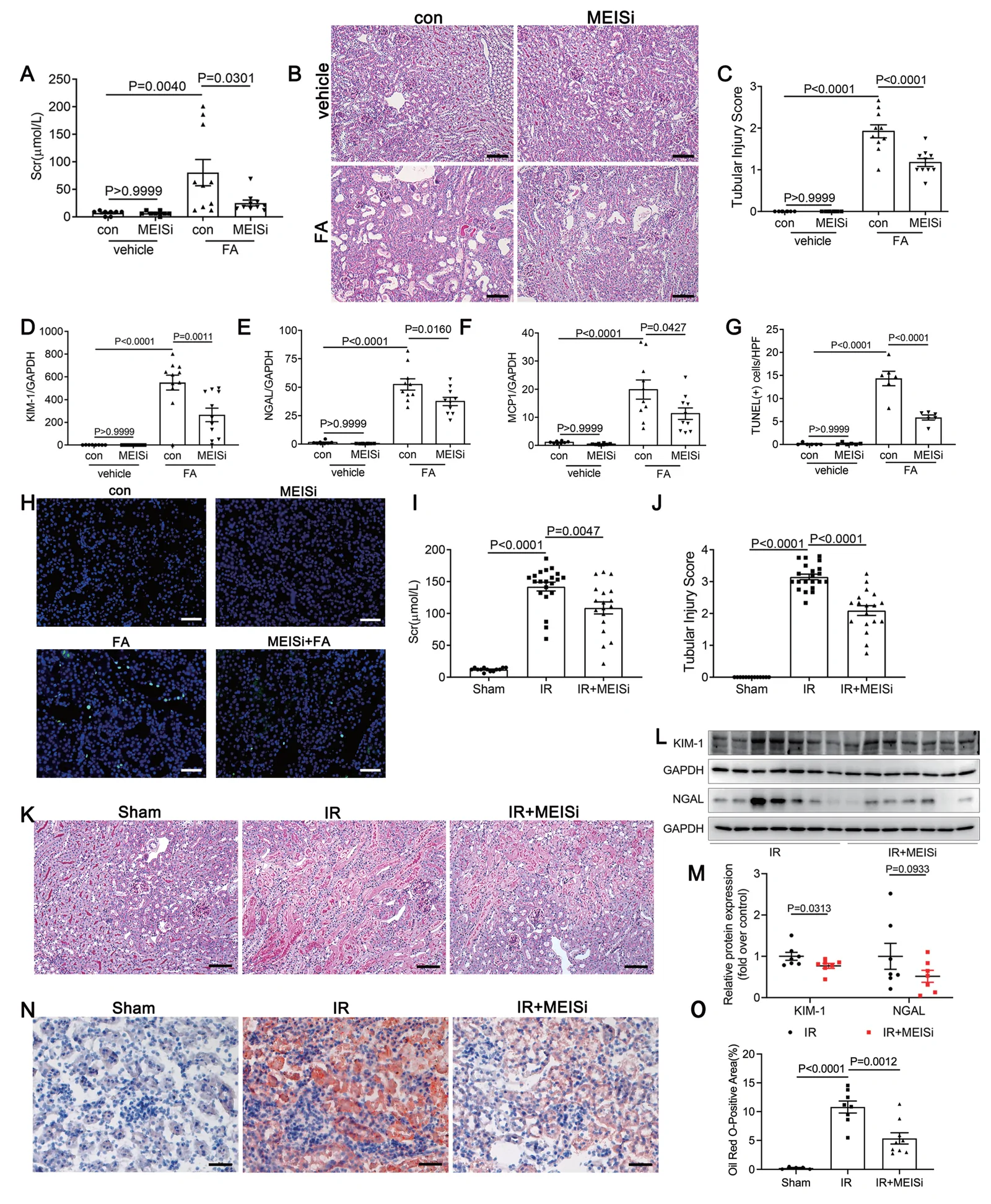
** Inhibition of Meis1 alleviates AKI induced by FA and IR.** Analysis of Scr after FA model treated with Meis1 inhibitor MEISi (n=8 in Vehicle group, n=10 in FA group). (**B**) Representative images for PAS staining after FA model treated with Meis1 inhibitor MEISi. Scale bar: 100μm. (**C**) Quantification of tubular injury score according to PAS staining (n=8 in Vehicle group, n=10 in FA group). (**D-F**) qRT-PCR analysis of KIM-1, NGAL and MCP-1 (n=8 in Vehicle group, n=10 in FA group). (**G** and **H**) Representative images and quantification for TUNEL staining after FA model treated with Meis1 inhibitor MEISi (n=6). Scale bar: 50μm. (**I**) Analysis of Scr after IR model treated with Meis1 inhibitor MEISi (we combined the results of two independent experiments, n=13 in Sham group, n=21 in IR group and n=18 in IR+MEISi group). (**J** and** K**) Representative images and quantification for PAS staining after IR model treated with Meis1 inhibitor MEISi (n=13 in Sham group, n=21 in IR group and n=18 in IR+MEISi group). Scale bar: 100μm. (**L** and **M**) Western blot and densitometric analysis for the expression of KIM-1 and NGAL after IR model treated with Meis1 inhibitor MEISi (n=7). (**N** and** O**) Representative images and quantification for Oil Red O staining of kidney tissues of IR model treated with Meis1 inhibitor MEISi (n=5 in Sham group, n=8 in IR group and n=9 in IR+MEISi group). Scale bar: 50μm.Data are presented as mean ± SEM. Data were statistically analyzed using unpaired two-tailed Student's t-test (**M**) or One-way ANOVA (**A**, **C**, **D**, **E**, **F**, **G**, **I**,** J** and **O**). The P-values were shown in the figures.

## Data Availability

The raw RNA-seq and RRBS data generated in this study have been deposited in the NCBI Sequence Read Archive (SRA) under accession number PRJNA1328626 and PRJNA1328756. A secure link for reviewers is available here: https://dataview.ncbi.nlm.nih.gov/object/PRJNA1328626?reviewer=qng28ibs926p02ik1u8lm56b0s and https://dataview.ncbi.nlm.nih.gov/object/PRJNA1328756?reviewer=gq6i3g0l7q2sb09vccoeo4n3pa. The non-targeted metabolomics have been deposited in the Metabolomics Workbench (Study ID: ST004295, https://dev.metabolomicsworkbench.org:22222/data/DRCCMetadata.php?Mode=Study&StudyID=ST004295&Access=CshA9766). All these datasets are publicly available as of the date of publication. All the data that support the findings of this study are available from the corresponding author upon reasonable request.

## Abbreviations

AKI: acute kidney injury
PTECs: proximal tubular epithelial cells
Upb1: β-ureidopropionase 1
3-UPA: 3-ureidopropionate
Meis1: myeloid ecotropic viral integration site 1
FA: folic acid
IR: ischemia/reperfusion injury
TCA cycle: tricarboxylic acid cycle
NIH: National Library of Medicine
KIT: Kidney Interactive Transcriptomics
DPYD: dihydropyrimidine dehydrogenase
DPYS: dihydropyrimidinase
TALE: three amino acid loop extension
HIF: hypoxia inducible factor
CKD: chronic kidney disease
FBS: fetal bovine serum
DMEM: Dulbecco's modified Eagle's medium
Upb1-KO: knockout
Meis1 cKI: renal proximal tubular-specific Meis1 conditional knock-in
MEISi: Meis1 inhibitor
Scr: serum creatinine
BUN: blood urea nitrogen
PAS: Periodic Acid-Schiff
TUNEL: terminal deoxynucleotidyl transferase-mediated dUTP nick end labeling
UHPLC-MS/MS: ultra-high performance liquid chromatography-tandem mass spectrometry
H/R: hypoxia/reoxygenation
RRBS: reduced representation bisulfite sequencing
IHC: immunohistochemistry
OCT: optimal cutting temperature
OCR: oxygen consumption rate
TEM: transmission electron microscopy
SDH: succinate dehydrogenase
ChIP: chromatin immunoprecipitation
CUT&RUN: cleavage under targets and release using nuclease
AKI-to-CKD: acute kidney injury to chronic kidney disease
UUO: unilateral ureteral obstruction
LC-MS/MS: liquid chromatography-tandem mass spectrometry
DEGs: differential expressed genes
KEGG: Kyoto Encyclopedia of Genes and Genomes
DMRs: differentially methylated regions
GO: gene ontology
DAG: directed acyclic graph
BPs: biological processes
FAO: fatty acid β-oxidation
ETC: electron transport chain
IOD: integrated optical density
LTL: lotus tetragonolobus lectin
ALT: alanine aminotransferase
AST: aspartate aminotransferase
CK-MB: creatine kinase-MB
LDH: lactate dehydrogenase
LUAD: lung adenocarcinoma
GC-MS: gas chromatography-mass spectrometry
