# Supplementary material for: Dysregulation of the Upb1-3-ureidopropionate Pathway Impairs Fatty Acid Metabolism and Exacerbates Acute Kidney Injury
Source: Int J Biol Sci. 2026 Aug 24;22(14):7644–66. doi: 10.7150/ijbs.135329 (PMC13617996; doi:10.7150/ijbs.135329)
Supplement: Supplementary file 1 [file ijbsv22p7644s1.pdf]

Supplementary materials

Supplemental Figures

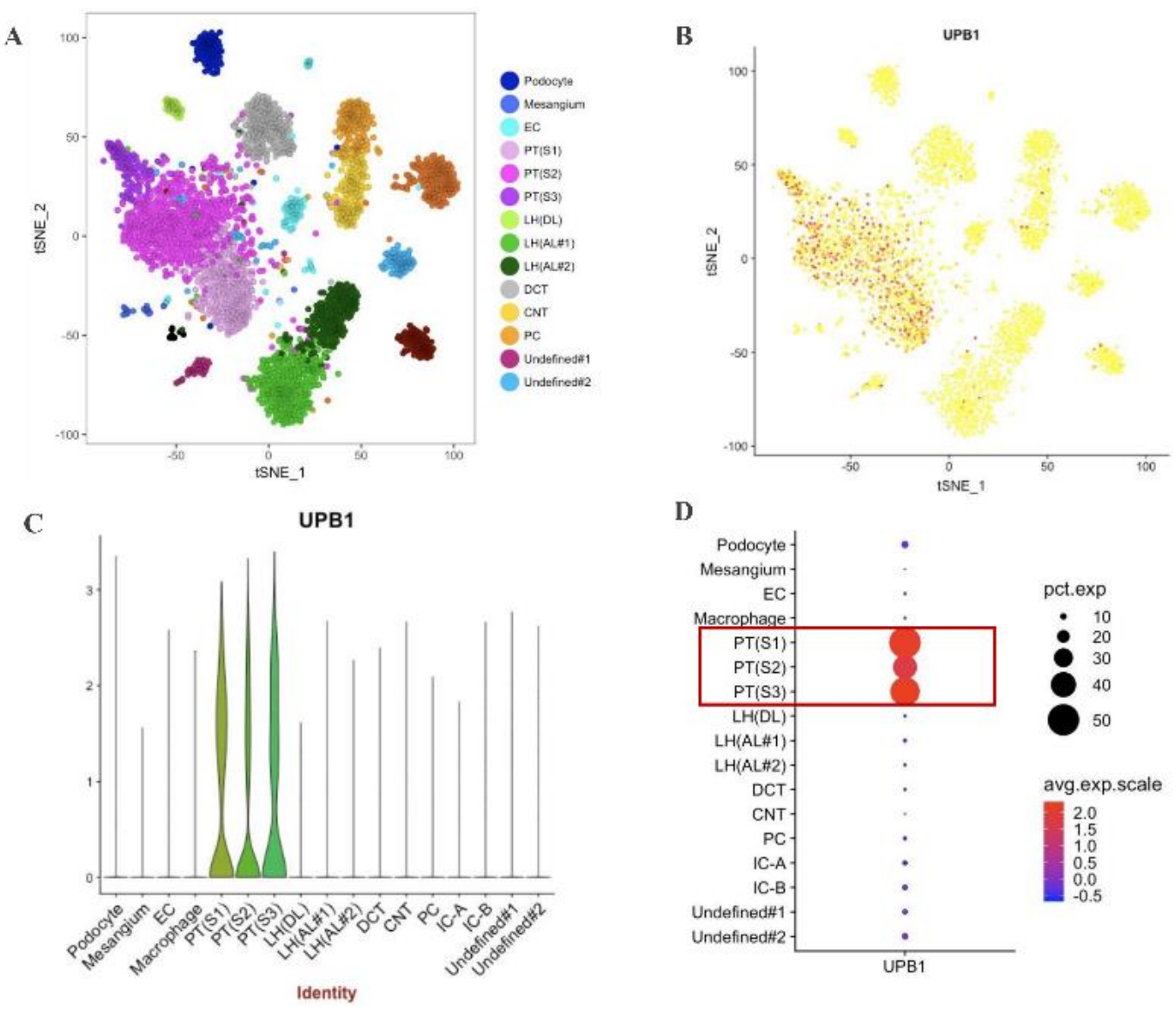

Figure S1. The expression of UPB1 in the Kidney Interactive Transcriptomics database.

(A-D) UPB1 is detectable across multiple renal cell populations in the Healthy Adult Kidney Dataset (PMID: 30449713), with its highest expression observed in proximal tubular epithelial cells.

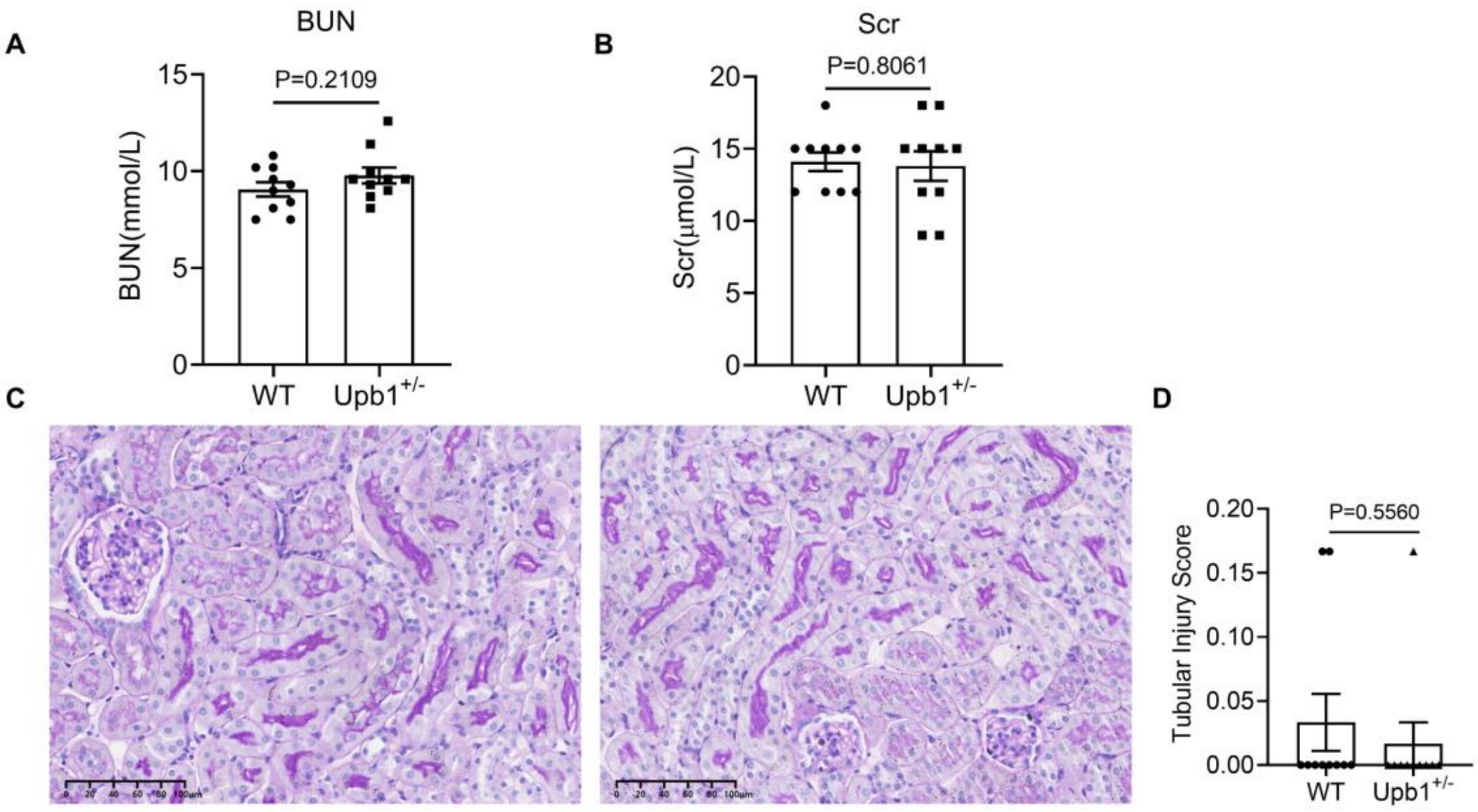

Figure S2. Baseline renal function and histology between untreated Upb1$^{+/-}$ and WT mice.

(**A**-**B**) Analysis of BUN and Scr under baseline conditions between untreated Upb1$^{+/-}$ and WT mice.

(C) Representative images for PAS staining of untreated Upb1$^{+/-}$ and WT mice. Scale bar: 100μm.

(**D**) Quantification of tubular injury score according to PAS staining of untreated Upb1$^{+/-}$ and WT mice (n=10 in each group).

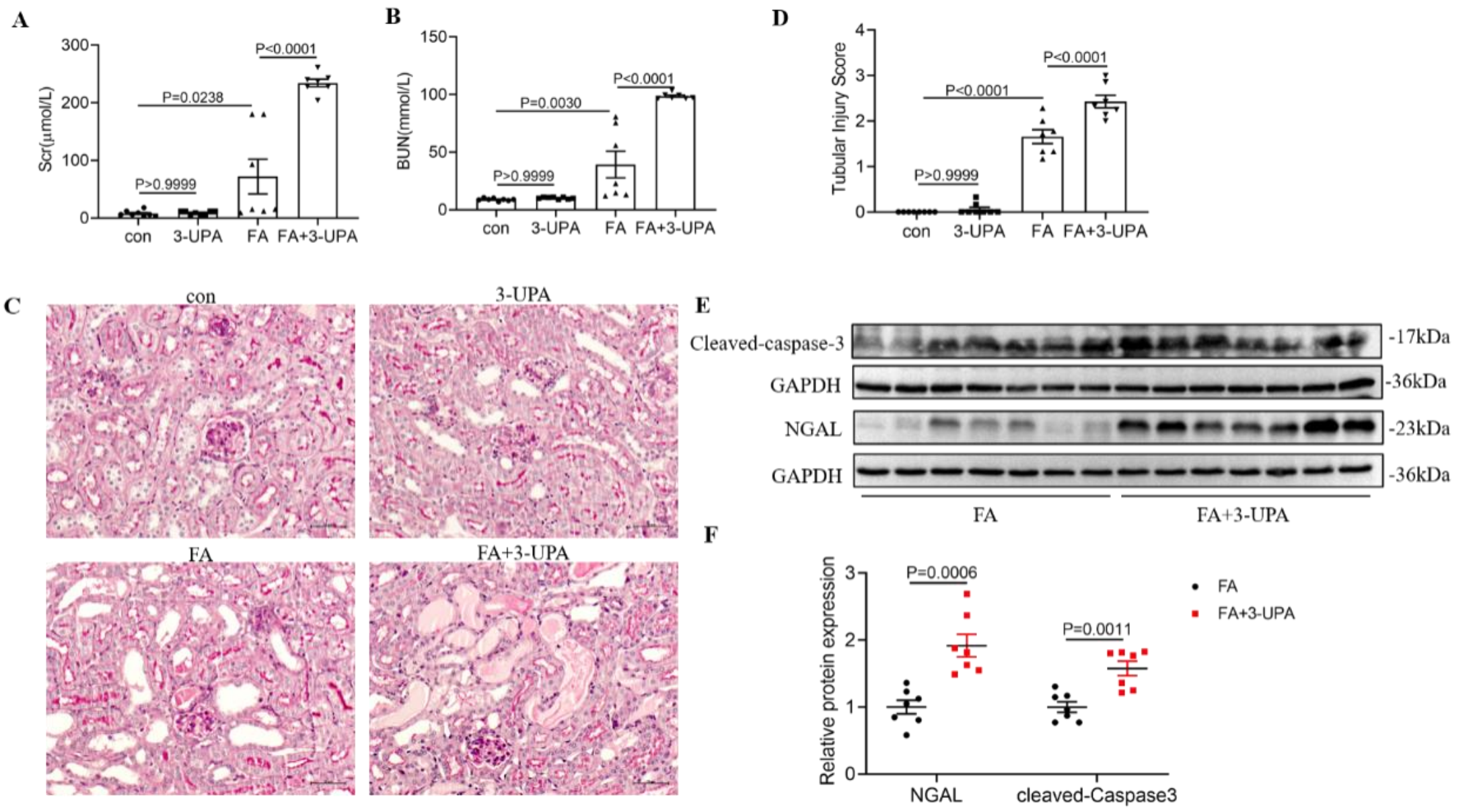

Figure S3. Administration of high concentration 3-UPA significantly aggravated AKI induced by folic acid.

(**A** and **B**) Analysis of serum creatinine (Scr) and blood urea nitrogen (BUN) after FA model in C57BL/6 mice treated with 3-UPA (260 mg/kg, twice daily for two days, intraperitoneally) (n=8 in con group or 3-UPA group, n=7 in FA group or FA+3-UPA group). The FA group initially comprised 10 mice, as did the FA+3-UPA group. However, three mice in the FA group failed to develop the model, while three mice in the FA+3-UPA group died. So, these mentioned animals were excluded from statistical analysis. One-way ANOVA. (**C**) Representative images for PAS staining of FA-induced AKI mice kidneys followed by 3-UPA treatment. Scale bar: 100μm. (**D**) Quantification of tubular injury score according to PAS staining of FA model in mice followed by 3-UPA treatment. (n=8 in con group or 3-UPA group, n=7 in FA group or FA+3-UPA group). One-way ANOVA. (**E** and **F**) Western blot and densitometric analysis for the expression of cleaved-caspase 3 and NGAL in FA-induced AKI mice kidneys treated with 3-UPA administration (n=7). Two-tailed Student's t-test. Data are presented as mean ± SEM. The P-values were shown in the figures.

A

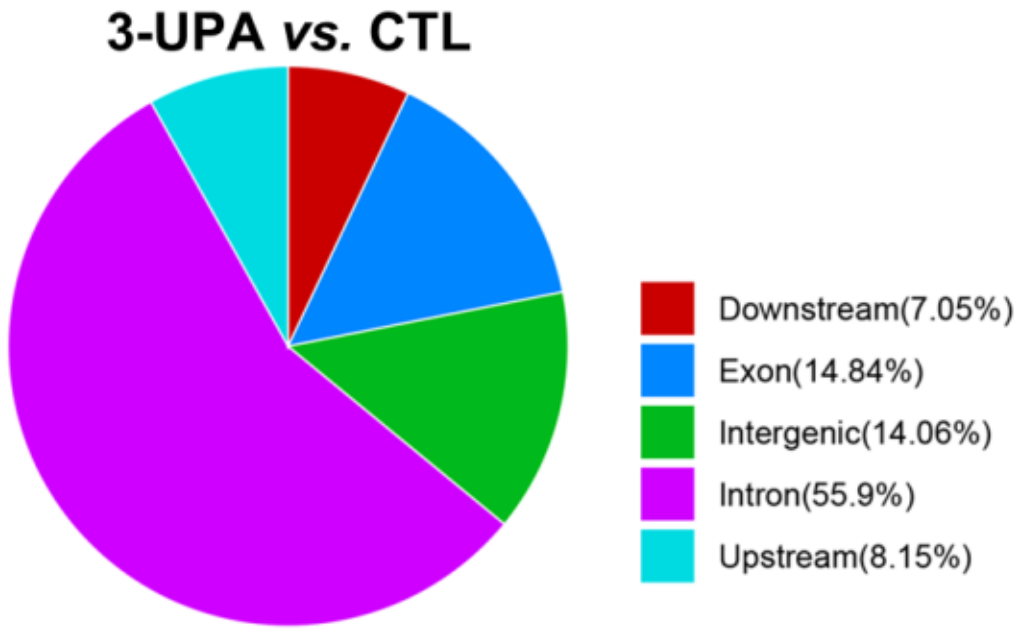

Distribution of DMRs

Group	3-UPA *vs.* CTL
Number of hypermethylated DMRs	1215
Number of hypomethylated DMRs	2621
Total number of DMRs	3836
Total gene number with DMRs	2446
Number of genes with DMR at upstream regulatory regions	448

B

KEGG Pathway Classification

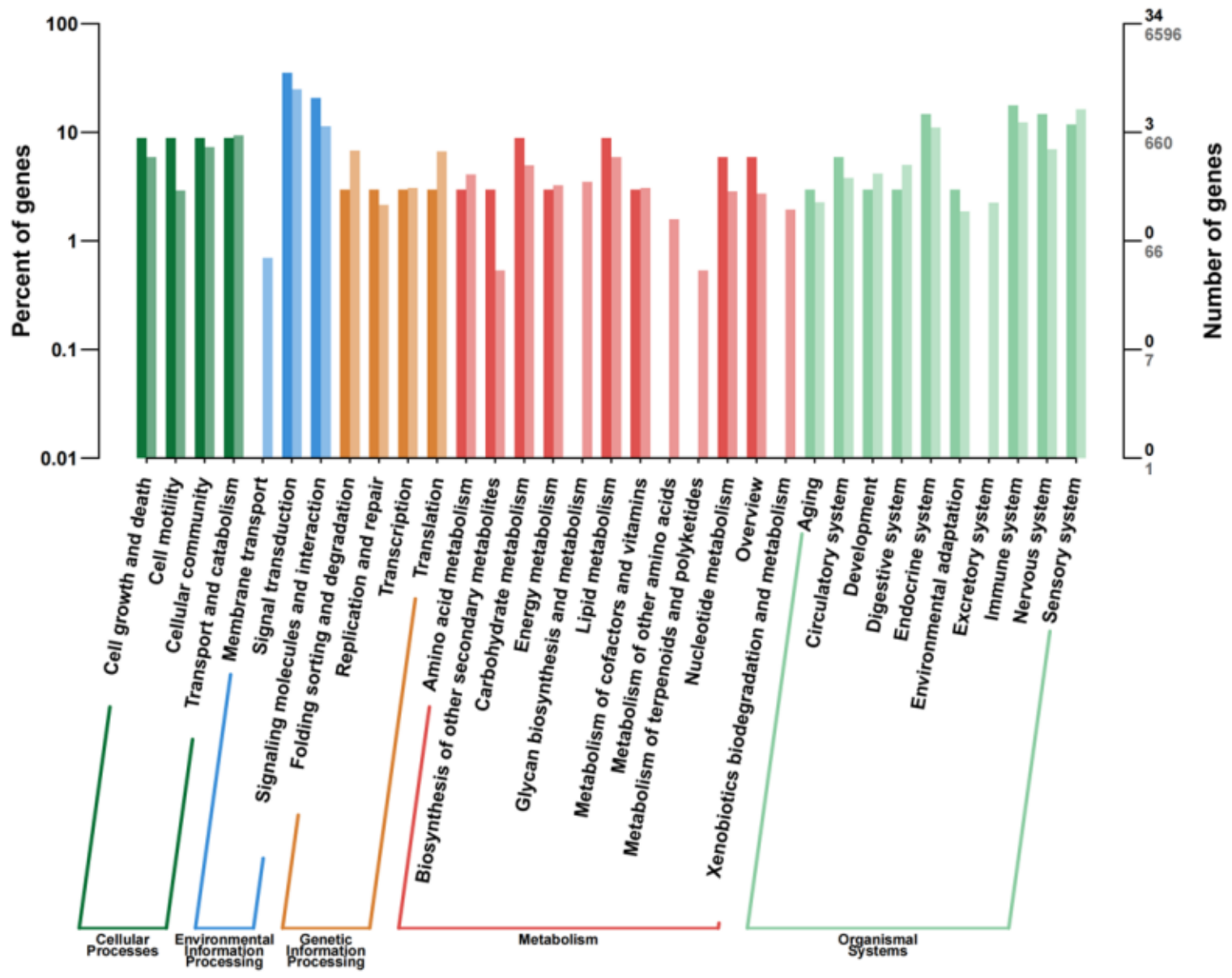

C

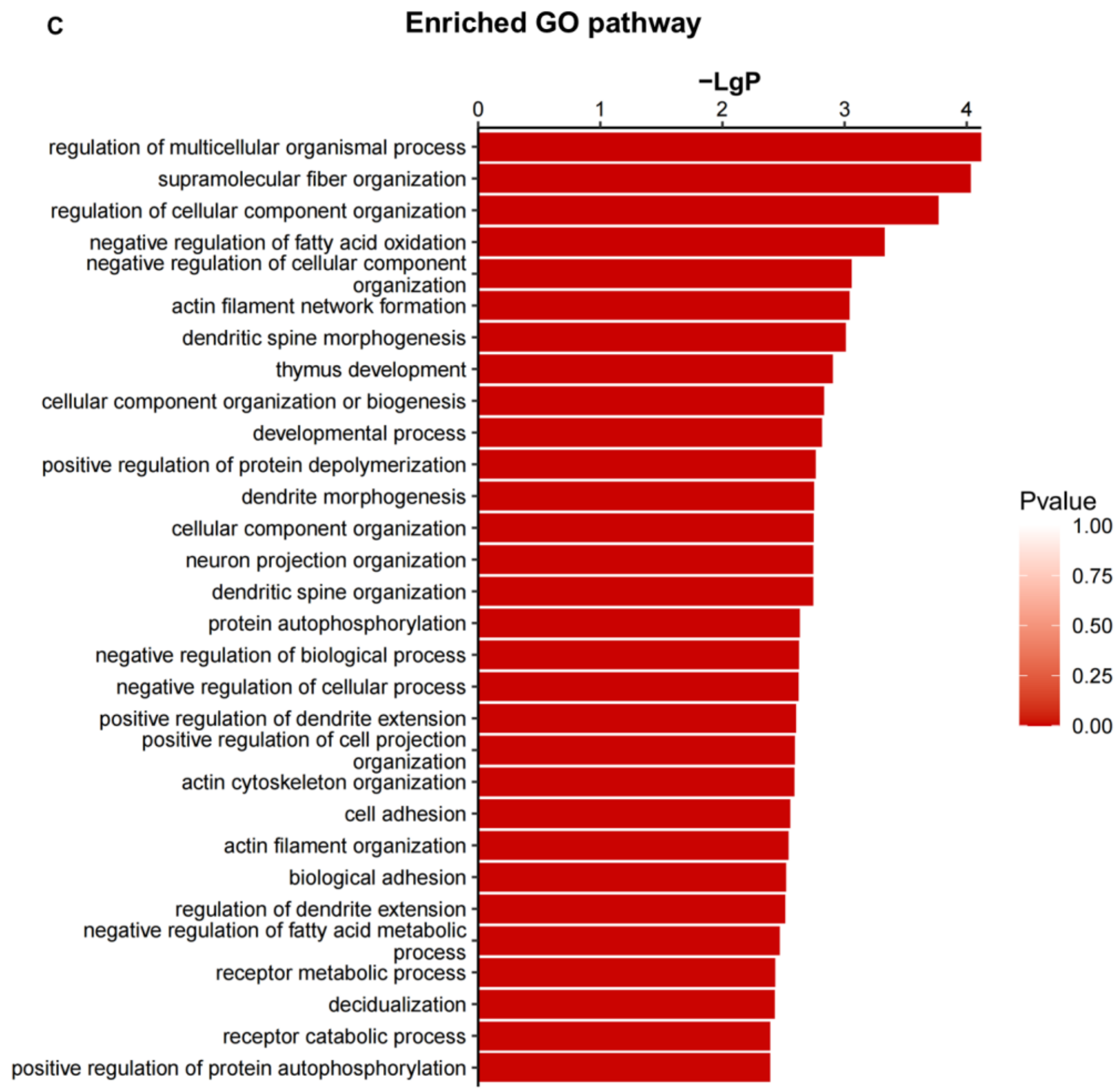
Enriched GO pathway
−LgP
0
1
2
3
4
regulation of multicellular organismal process
supramolecular fiber organization
regulation of cellular component organization
negative regulation of fatty acid oxidation
negative regulation of cellular component organization
actin filament network formation
dendritic spine morphogenesis
thymus development
cellular component organization or biogenesis
developmental process
positive regulation of protein depolymerization
dendrite morphogenesis
cellular component organization
neuron projection organization
dendritic spine organization
protein autophosphorylation
negative regulation of biological process
negative regulation of cellular process
positive regulation of dendrite extension
positive regulation of cell projection organization
actin cytoskeleton organization
cell adhesion
actin filament organization
biological adhesion
regulation of dendrite extension
negative regulation of fatty acid metabolic process
receptor metabolic process
decidualization
receptor catabolic process
positive regulation of protein autophosphorylation
Pvalue
1.00
0.75
0.50
0.25
0.00

D

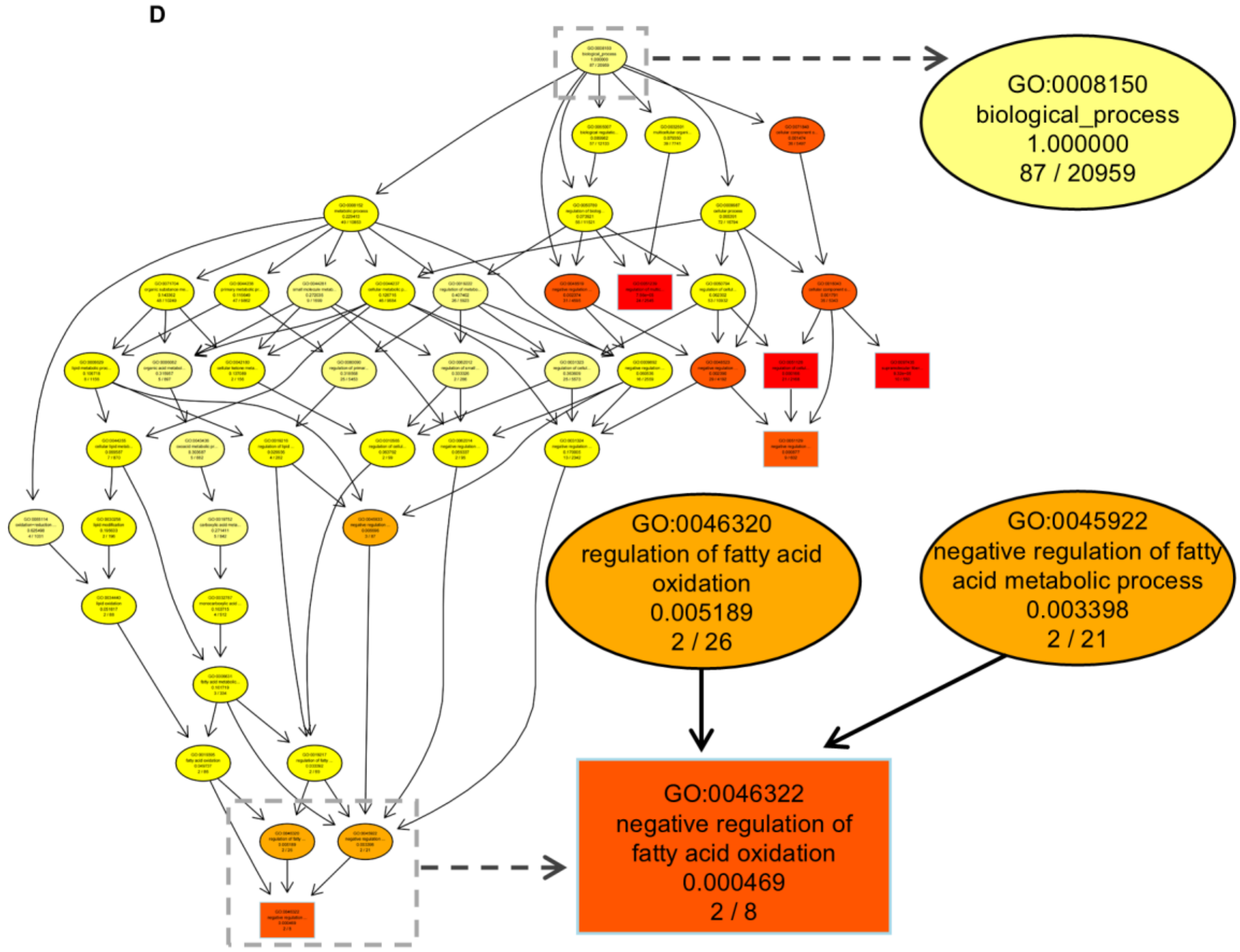
GO:0008150
biological_process
1.000000
87 / 20959
GO:0046320
regulation of fatty acid oxidation
0.005189
2 / 26
GO:0045922
negative regulation of fatty acid metabolic process
0.003398
2 / 21
GO:0046322
negative regulation of fatty acid oxidation
0.000469
2 / 8

Figure S4. Enlarged versions of all key RRBS results of Figure 4.

(**A**) Location and numbers of differentially methylated regions (DMRs) from the RRBS analysis of TKPTs treated with 3-UPA (10mM) for 24 hours (n=3). (**B**) KEGG pathway classification results of hypermethylated DMRs in the 3-UPA group *vs.* control group. (**C**) GO enrichment pathway analysis of hypermethylated DMRs in the 3-UPA group *vs.* control group. (**D**) The directed acyclic graph (DAG), serving as a graphical display of the GO enrichment analysis outcomes, emphasized the BPs significantly modulated by 3-UPA.

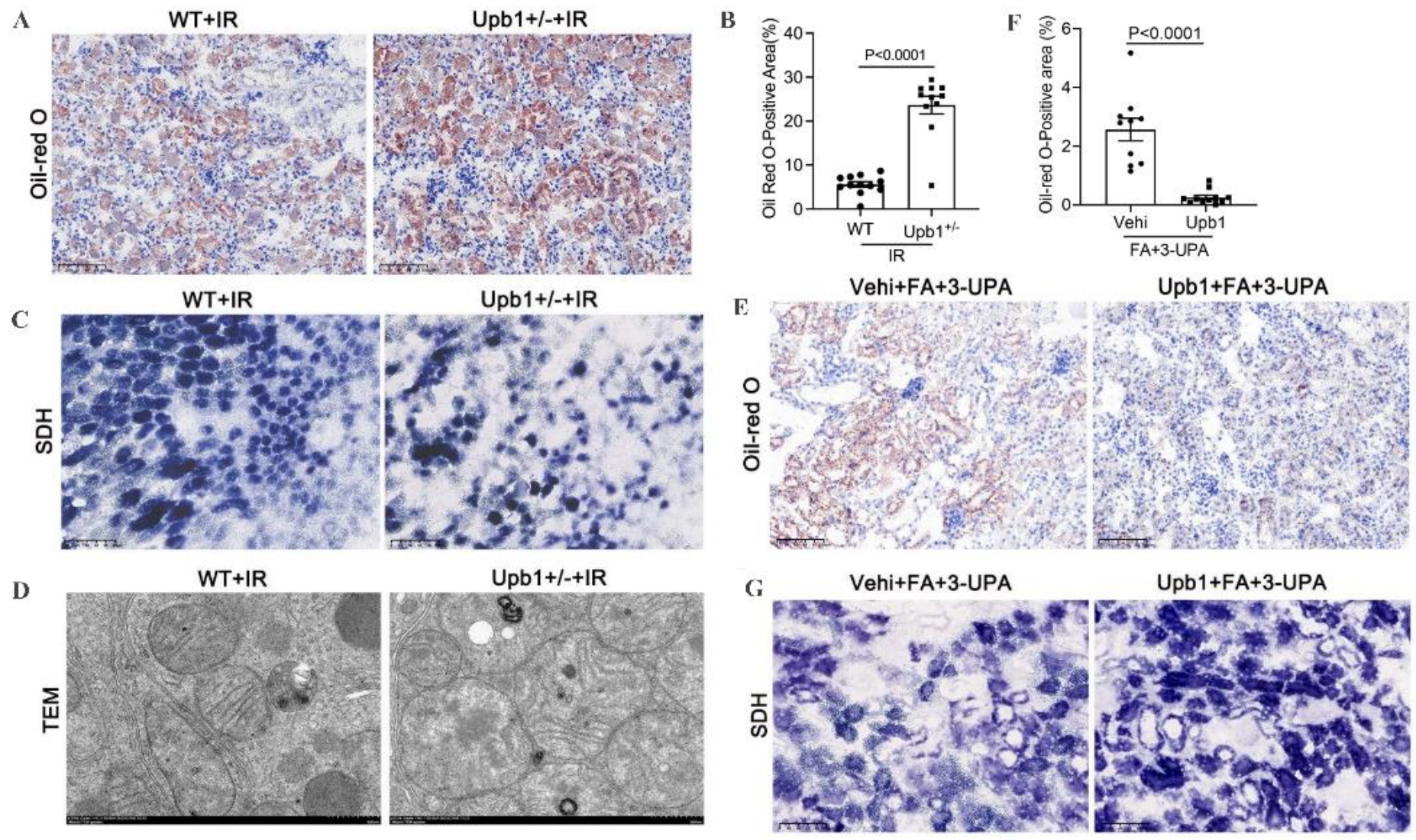

Figure S5. Oil red O staining, SDH staining, and TEM images of kidneys from AKI mice with Upb1 knockdown or overexpression.

(**A** and **B**) Representative images and quantification for Oil Red O staining of kidney tissues of Upb1 $^{+/-}$ mice and WT mice in IR-AKI model (n=12). Scale bar: 100μm.Two-tailed Student's t-test. (**C**) Representative images for SDH staining of kidney tissues of Upb1 $^{+/-}$ mice and WT mice in IR-AKI model. Scale bar: 100μm. (**D**) Representative transmission electron microscopy (TEM) images for mitochondria of proximal tubular epithelial cells in Upb1 $^{+/-}$ mice and WT mice after IR. Scale bar: 500nm. (**E** and **F**) Representative images and quantification for Oil Red O staining of kidney tissues of Upb1 overexpressed mice followed by 3-UPA treatment after FA model (n=10). Scale bar: 100μm.Two-tailed Student's t-test. (**G**) Representative images for SDH staining of kidney tissues of Upb1 overexpressed mice followed by 3-UPA treatment after FA model. Scale bar: 100μm. Data are presented as mean ± SEM. The P-values were shown in the figures.

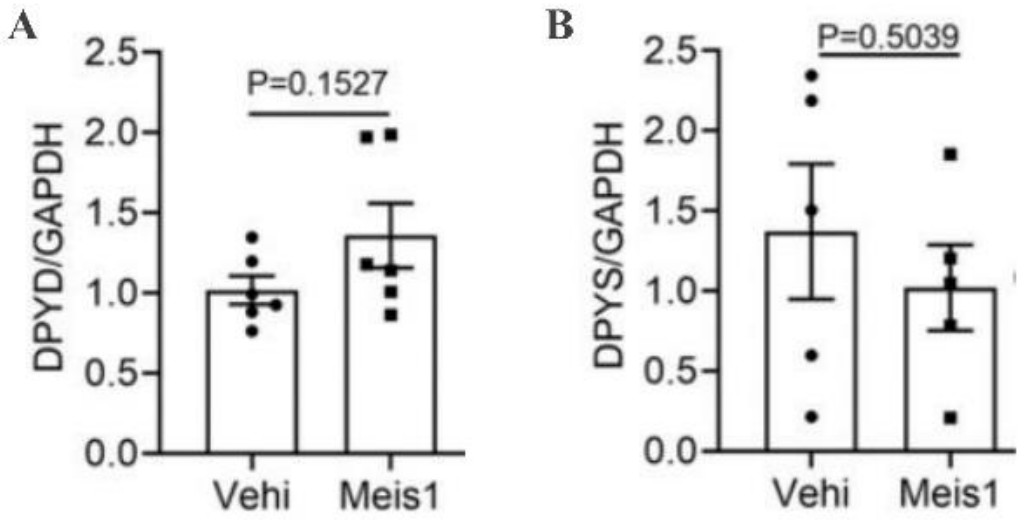

Figure S6. The expression of DPYD and DPYS of TKPTs after overexpressing Meis1 in vitro. (**A**-**B**) qRT-PCR analysis of DPYD and DPYS, two key enzymes of the 3-UPA metabolic pathway in TKPTs after transfection with Meis1 overexpression plasmid for 24h (n=6). Two-tailed Student's t-test. Data are presented as mean ± SEM. The P-values were shown in the figures.

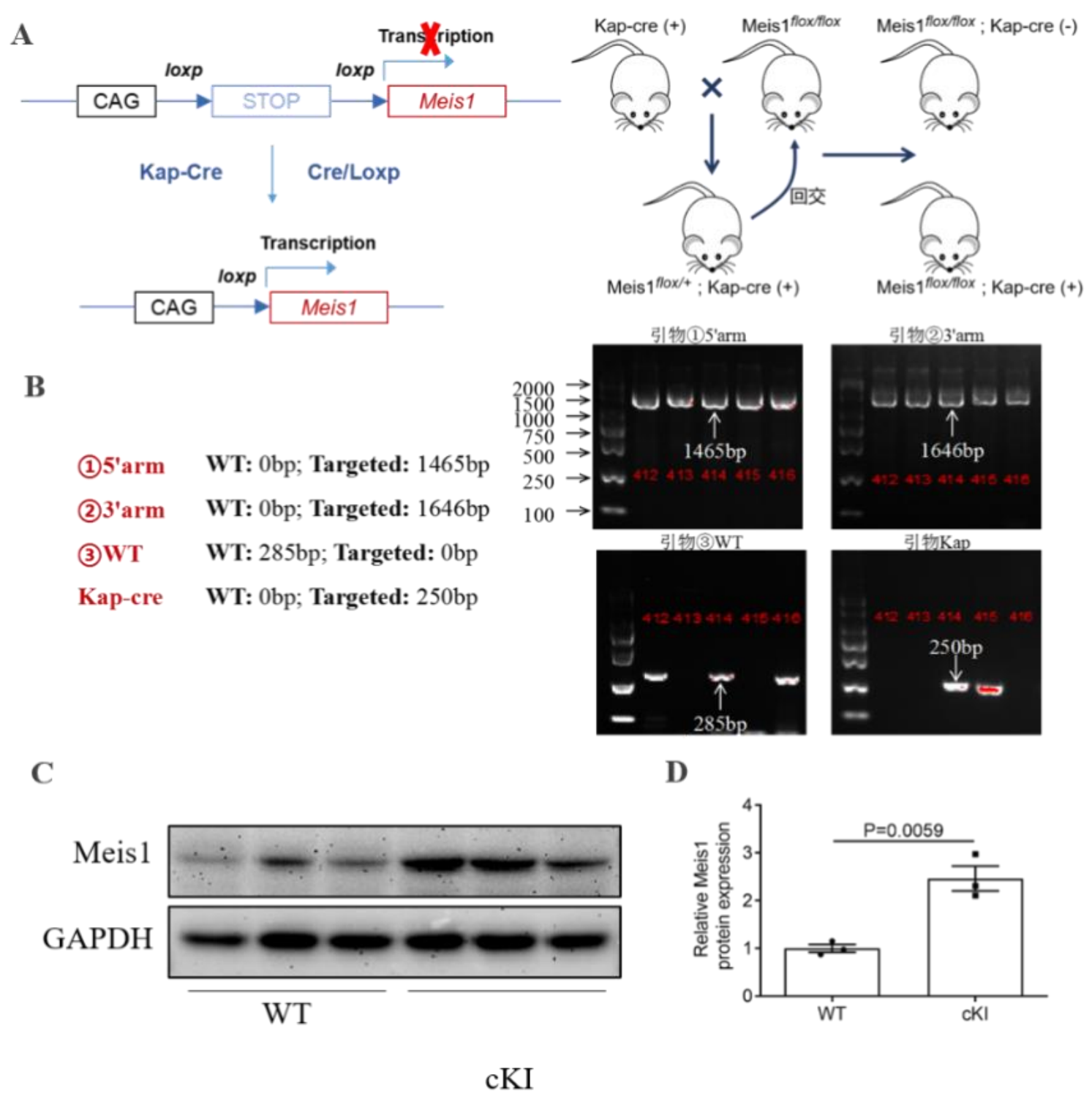

Figure S7. Generation and expression verification of Meis1 cKI mouse with specific overexpression of renal proximal tubule epithelial cells.

(**A**) Schematic diagram of generation of Meis1 conditional knock-in mice (cKI) mouse with specific overexpression of renal proximal tubule epithelial cells using the Cre-loxP recombinase system. (**B**) Genotype identification of Meis1 cKI mice using polymerase chain reaction (PCR). (**C** and **D**) Western blot for the expression of Meis1 in cKI mice after isolation of renal tubules (n=3). Two-tailed Student's t-test. Data are presented as mean ± SEM. The P-value was shown in the figure.

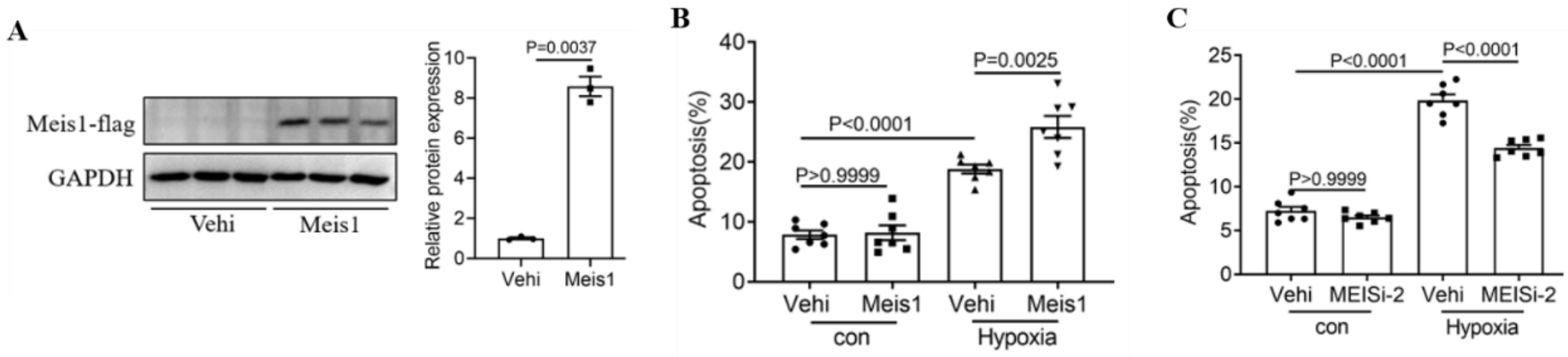

Figure S8. Meis1 demonstrated the capacity to increase of cell apoptosis in vitro.

(**A**) Western blot and densitometric analysis for the expression of Meis1 in cultured TKPTs after transfection with Meis1 plasmid for 48h (n=3). Two-tailed Student's t-test. (**B**) Quantification of apoptosis of TKPTs after transfection with Meis1 plasmid followed by normal conditions or hypoxia (1%O_2) culture for 24h/reoxygenation for 2h (n=7). One-way ANOVA. (**C**) Quantification of apoptosis of TKPTs after treated with Meis1 inhibitor MEISi followed by normal conditions or hypoxia (1%O_2) culture for 24h/reoxygenation for 2h (n=7). One-way ANOVA. Data are presented as mean ± SEM. The P-values were shown in the figures.

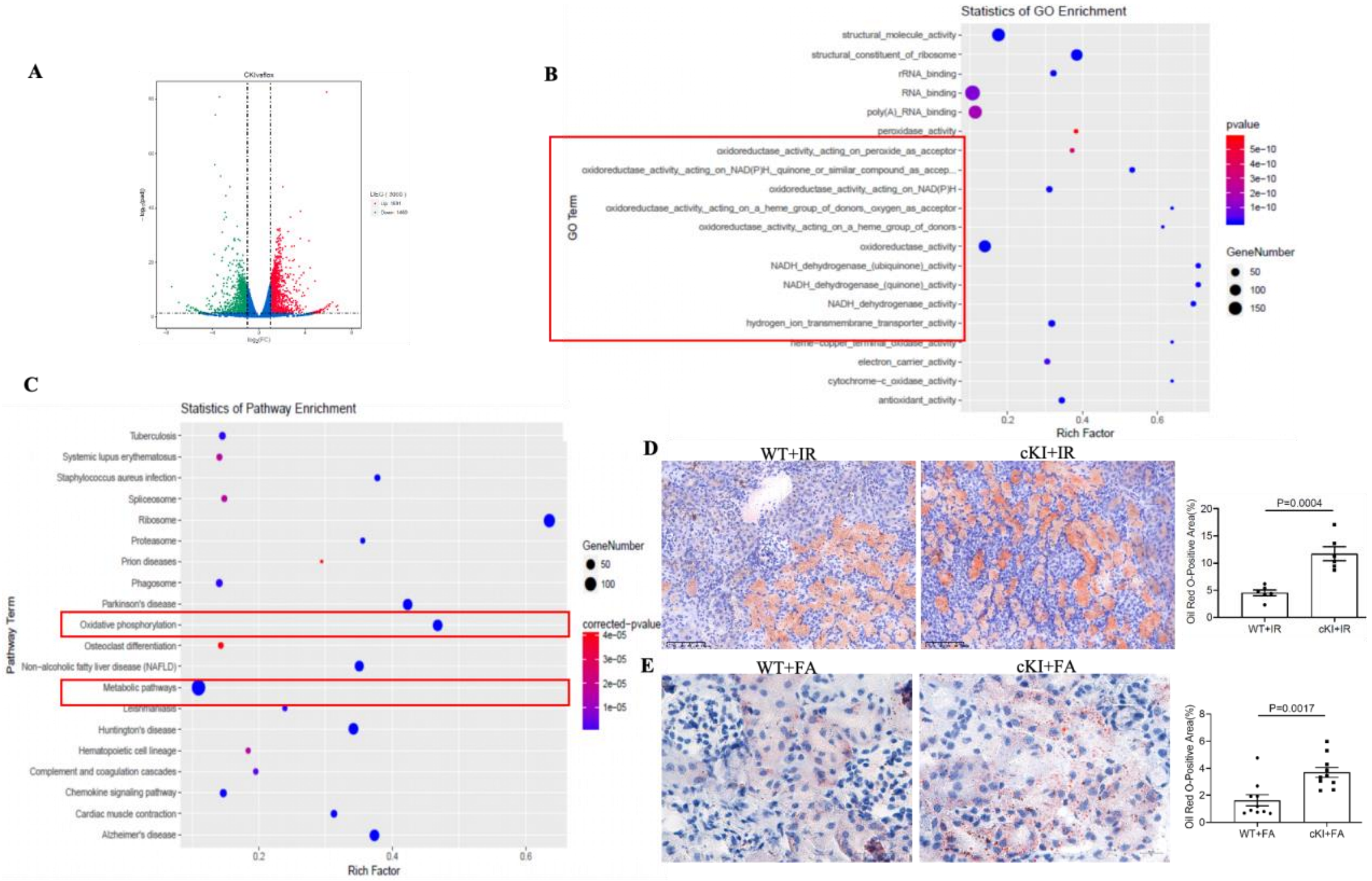

Figure S9. Proximal tubular specific overexpression of Meis1 interferes with fatty acid oxidative (FAO) metabolism in tubular epithelial cells.

(**A**) Volcano-plot of differential expressed genes (DEGs) from the RNA-sequencing analysis of renal tubules from Meis1-cKI and WT mice (n=3). (**B** and **C**) Bar graphs of GO enrichment and KEGG pathway analysis results of DEGs from the RNA-seq data. GO enrichment analysis indicated that these DEGs were predominantly enriched in the "oxidoreductase activity", and the pathways enrichment was also related to "Oxidative phosphorylation" and "Metabolic pathways". (**D** and **E**) Representative images for Oil Red O staining of kidney tissues of IR- and FA- induced AKI models using Meis1-cKI and WT mice. Scale bar: 100μm (IR model) or 20μm (FA model). Quantification of Oil Red O staining positive area of kidney tissues in IR- induced AKI model (n=6) and FA-induced AKI model (n=10). Two-tailed Student's t-test. Data are presented as mean ± SEM. The P-values were shown in the figures.

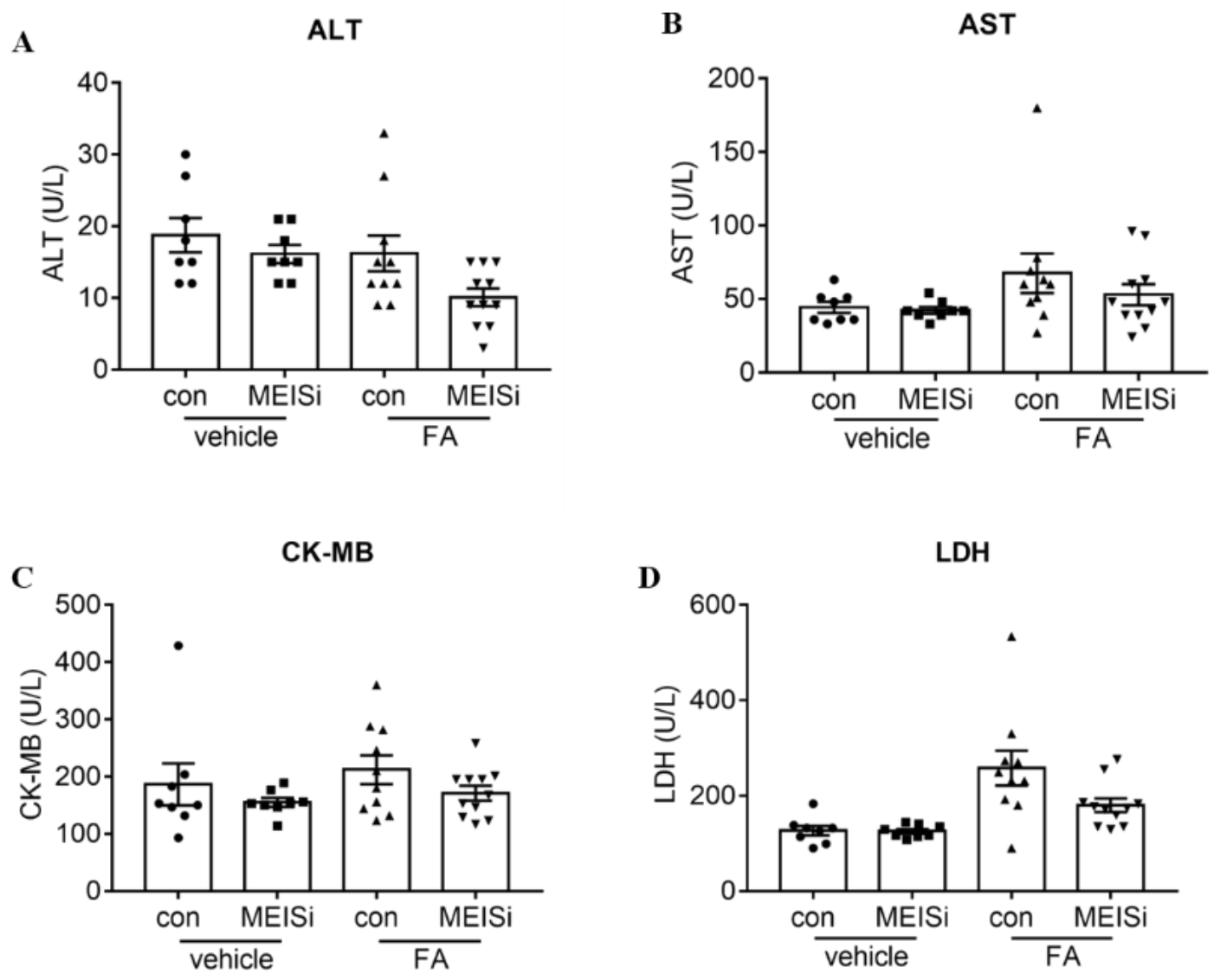

Figure S10. Therapeutic doses of Meis1 inhibitor MEISi had no obvious hepatic, heart, and systemic toxicity.

(A-D) Analysis of serum alanine aminotransferase (ALT), aspartate aminotransferase (AST), creatine kinase-MB (CK-MB) and lactate dehydrogenase (LDH) after FA model treated with Meis1 inhibitor MEISi (n=8 in Vehicle group, n=10 in FA group). The values represent means ± SEM. One-way ANOVA.

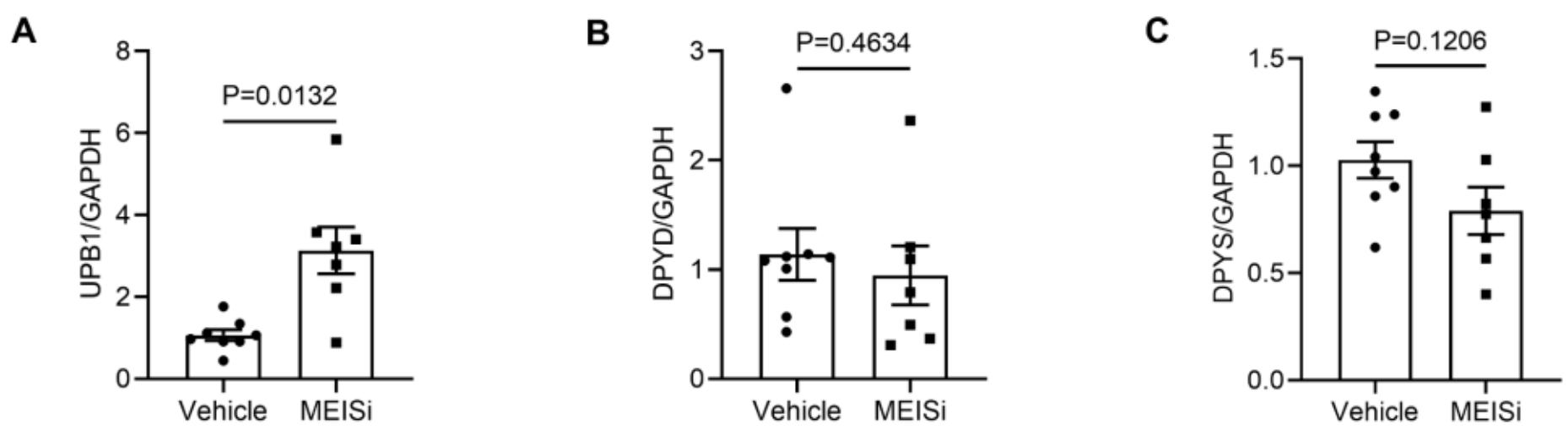

Figure S11. The expression of UPB1, DPYD and DPYS of C57BL/6J mice kidneys treated with Meis1 inhibitor MEISi.

(**A**-**C**) qRT-PCR analysis of UPB1, DPYD and DPYS, three key enzymes of the 3-UPA metabolic pathway in C57BL/6J mice kidneys treated with Meis1 inhibitor MEISi (n=8 in Vehicle group, n=7 in MEISi group). Two-tailed Student's t-test. Data are presented as mean ± SEM. The P-values were shown in the figures.

Table S1. The basic information and diagnosis of AKI patients

Sex	Age(yr)	Pathological diagnosis	Scr (μmol/L)	BUN (mmol/L)
Male	2.4	acute tubular necrosis	65.2	5.30
Female	12.0	acute tubular necrosis	110.0	10.02
Male	1.0	acute tubular necrosis; interstitial nephritis	290.0	9.04
Female	1.1	acute interstitial nephritis	548.0	18.73
Male	8.0	acute tubular necrosis	616.0	26.50
Male	0.5	acute tubular necrosis	334.0	20.40
Female	10.3	acute interstitial nephritis	75.0	5.60
Female	13.8	acute interstitial nephritis	329.0	4.25
Female	1.0	acute interstitial nephritis	245.0	11.90
Female	0.3	acute tubular necrosis	76.0	4.89

Table S2. The sequences of the primers used in the study

Primer Name	Primer Sequence 5′-3′
Mouse-GAPDH	F：GTCTTCACTACCATGGAGAAGG
	R：TCATGGATGACCTTGGCCAG
Mouse-MCP1	F：GCTCTCTCTTCCTCCACCAC
	R：ACAGCTTCTTTGGGACACCT
Mouse-TNFα	F：GGAGCTGGCTACTTCTCGC
	R：GGGAACATCCTCCTTCAACAG
Mouse-KIM-1	F：ACATATCGTGGAATCACAACGAC
	R：ACTGCTCTTCTGATAGGTGACA
Mouse-NGAL	F：CAAGGAGCTGTCCCCTGAAC
	R：TGAACCATTGGGTCTCTGCG
Mouse-Upb1	F：TGGCAGGATTGCAGTGAACA
	R：GTGATTGGCAATGGCTGCAT
Mouse-Bax	F：TGGAGCTCAACCACATTAGCA
	R：GATCAGCTCGGGCACTTTAG
Mouse-Meis1	F：GCAAAGTATGCCAGGGGAGTA
	R：TCCTGTGTTAAGAACCGAGGG
Mouse-DPYD	F：GACATCGAGAGTATCCTGGCT
	R：AGGTAAAGCAGTTCTTGTCCG
Mouse-DPYS	F：CCACAGGGACGACTTCTCATC
	R：CGCTGCATCTAGGATTCGCA

Table S3. Sequences of ChIP primers

Primer Name	Primer Sequence 5′-3′
-711~-705	F：GCTTCTTTGCTCCTGAAGGGA
	R：TATGGGCACTTTCCTGCGGTA
-441~-435	F：CCCCTCGGGGGTTAAAACT
	R：TCCCTCACAGGACTACCGAG
-317~-311	F：CTCGGTAGTCCTGTGAGGGA
	R：CGGGTCTGAGGTAAACGGC
GAPDH promoter	F: CTATCATCCGATCTGACCATCACG
	R: CGCCCGACGCTTCTGGGAGTTGTA

Table S4. Sequences of CUT&RUN primers

Primer Name	Primer Sequence 5′-3′
-870~-856	F：CAAAACGGGCACAACTTGGA
	R：GTGTGGGAGACTTCACAAAGC
-774~-760	F：TCTCCCACACAAATCTCTCAGC
	R：ACCACTTGCCGCAGTTTAGT
+54~+68	F：GAACCCTCCTCACAGAGCAC
	R：TGGTTGCAGGGACTGATGTC
